# *Neofusicoccum parvum* Colonization of the Grapevine Woody Stem Triggers Asynchronous Host Responses at the Site of Infection and in the Leaves

**DOI:** 10.3389/fpls.2017.01117

**Published:** 2017-06-28

**Authors:** Mélanie Massonnet, Rosa Figueroa-Balderas, Erin R. A. Galarneau, Shiho Miki, Daniel P. Lawrence, Qiang Sun, Christopher M. Wallis, Kendra Baumgartner, Dario Cantu

**Affiliations:** ^1^Department of Viticulture and Enology, University of California, DavisDavis, CA, United States; ^2^Department of Plant Pathology, University of California, DavisDavis, CA, United States; ^3^Department of Agriculture and Forest Science, Faculty of Life and Environmental Science, Shimane UniversityMatsue, Japan; ^4^Department of Biology, University of WisconsinStevens Point, WI, United States; ^5^United States Department of Agriculture-Agricultural Research Service, San Joaquin Valley Agricultural Sciences CenterParlier, CA, United States; ^6^United States Department of Agriculture-Agricultural Research Service, Crops Pathology and Genetics Research UnitDavis, CA, United States

**Keywords:** botryosphaeria dieback, wood decay, local defense response, systemic defense response, stilbenoids

## Abstract

Grapevine trunk diseases cause important economic losses in vineyards worldwide. *Neofusicoccum parvum*, one of the most aggressive causal agents of the trunk disease Botryosphaeria dieback, colonizes cells and tissues of the grapevine wood, leading to the formation of an internal canker. Symptoms then extend to distal shoots, with wilting of leaves and bud mortality. Our aim was to characterize the transcriptional dynamics of grapevine genes in the woody stem and in the leaves during *Neofusicoccum parvum* colonization. Genome-wide transcriptional profiling at seven distinct time points (0, 3, and 24 hours; 2, 6, 8, and 12 weeks) showed that both stems and leaves undergo extensive transcriptomic reprogramming in response to infection of the stem. While most intense transcriptional responses were detected in the stems at 24 hours, strong responses were not detected in the leaves until the next sampling point at 2 weeks post-inoculation. Network co-expression analysis identified modules of co-expressed genes common to both organs and showed most of these genes were asynchronously modulated. The temporal shift between stem vs. leaf responses affected transcriptional modulation of genes involved in both signal perception and transduction, as well as downstream biological processes, including oxidative stress, cell wall rearrangement and cell death. Promoter analysis of the genes asynchronously modulated in stem and leaves during *N. parvum* colonization suggests that the temporal shift of transcriptional reprogramming between the two organs might be due to asynchronous co-regulation by common transcriptional regulators. Topology analysis of stem and leaf co-expression networks pointed to specific transcription factor-encoding genes, including WRKY and MYB, which may be associated with the observed transcriptional responses in the two organs.

## Introduction

Grapevine trunk diseases cause important economic losses in vineyards worldwide (Hofstetter et al., 2012; Fontaine et al., 2016; Kaplan et al., 2016). The trunk diseases Esca, Eutypa dieback, Botryosphaeria dieback, and Phomopsis dieback, are caused by taxonomically unrelated fungi that colonize and kill woody tissue, while causing a variety of symptoms in the growing green tissues (Larignon et al., 2009; Bertsch et al., 2013; Bruez et al., 2014). To date, no grape species, either cultivated or wild, is known to be resistant (Surico et al., 2006; Wagschal et al., 2008; Larignon et al., 2009). Disease management relies on preventative practices, such as delayed pruning and application of pruning-wound protectants (Weber et al., 2007; Rolshausen et al., 2010) and post-infection removal of infected wood, followed by vine retraining (Sosnowski et al., 2011).

*Neofusicoccum parvum* (Pennycook & Samuels) is one of the most aggressive causal agents of Botryosphaeria dieback (Úrbez-Torres and Gubler, 2009). *N. parvum* colonizes the woody tissue through wounds (e.g., pruning wounds), causing internal cankers in the permanent woody-structure of the vine (i.e., spurs, cordons, and trunk). The infection also causes foliar chlorosis and necrosis, but more often what appears in the vineyard is “dieback,” the death of shoots and buds distal to the wood canker (Larignon et al., 2001; Úrbez-Torres, 2011). Because *N. parvum* and other grapevine trunk pathogens have not been isolated from the leaves of infected plants (Larignon and Dubos, 1997; Mugnai et al., 1999), it has been hypothesized that foliar symptoms are caused by extracellular compounds produced by the pathogens at the site of infection (Mugnai et al., 1999). Phytotoxic compounds could either be translocated to the leaves through the transpiration stream or induce a reaction cascade leading to the expression of symptoms in distal tissues (Mugnai et al., 1999). *N. parvum* was shown to produce a variety of phytotoxins *in vitro* (Andolfi et al., 2011; Bénard-Gellon et al., 2015). These compounds belong to different chemical classes, including but not limited to dihydrotoluquinones, epoxylactones, dihydroisocoumarins, hydroxybenzoic acids, and fatty esters (Evidente et al., 2010; Abou-Mansour et al., 2015; Uranga et al., 2016). The dihydrotoluquinones terremutin and mullein were detected in wood from grapevines with Botryosphaeria dieback symptoms and were shown to cause severe necrosis when applied to leaf disks (Abou-Mansour et al., 2015).

The genome of *N. parvum* encodes a large repertoire of putative virulence factors, potentially involved in primary and secondary plant cell wall decomposition and in the biosynthesis of secondary metabolites, including phytotoxins (Blanco-Ulate et al., 2013a; Massonnet et al., 2016). Comparative analysis with the genomes of multiple trunk pathogens indicated a significant expansion of gene families associated with specific oxidative functions in the *N. parvum* genome, which may contribute to lignin degradation and toxin biosynthesis (Morales-Cruz et al., 2015). Analysis of the *N. parvum* transcriptome during colonization of grapevine woody stems showed distinctive patterns of transcriptional induction and repression of specific cell wall-degrading and secondary metabolism functions, suggesting that *N. parvum* virulence activities vary at the different stages of fungal development and host colonization (Massonnet et al., 2016).

Plant tolerance to biotic stress has been extensively studied in model organisms, such as *Arabidopsis thaliana* and *Nicotiana* spp. (Jones and Dangl, 2006; Onaga and Wydra, 2016). Nonetheless, pathogen responses in woody tissues of perennial plants are much less understood (Tobias and Guest, 2014). Wood infection has been described to lead to: (i) physical responses, such as the formation of tyloses and gels in xylem vessels and of traumatic resin ducts in secondary phloem (Franceschi et al., 2005; Sun et al., 2006; Yadeta and Thomma, 2013), (ii) transcriptomic reprogramming in xylem parenchyma cells, consisting of significant changes in the expression of genes associated with defense, detoxification or redox processes, cell wall biosynthesis, hormone signaling and secondary metabolism (Camps et al., 2010; Barakat et al., 2012; Xu et al., 2013; Mangwanda et al., 2015), and (iii) metabolic changes with accumulation of different proteins and secondary metabolites in the xylem sap and phloem parenchyma, including pathogenesis-related (PR) proteins, peroxidases, superoxide dismutases, glutathione *S*-transferases, proteases, xyloglucan-endotransglycosylases, phenols, phytoalexins, and lignin-like compounds (Franceschi et al., 2005; Wallis et al., 2008; Yadeta and Thomma, 2013; Spagnolo et al., 2014).

Local responses to pathogen infection can lead to the activation of defense reactions in other parts of the plant not colonized by the pathogen (i.e., distal or systemic responses; Heil and Bostock, 2002). Our limited understanding of distal responses to trunk pathogens in grapevine confounds detection efforts. Camps et al. (2010) analyzed the systemic responses to *Eutypa lata* infection in both symptomatic and asymptomatic leaves. They reported the up-regulation of genes involved in defense reactions in symptomatic leaves, while the lack of foliar symptoms was associated with the up-regulation of photosynthesis-related genes. By studying the transcriptomic dynamics in grape leaves after *N. parvum* infection, Czemmel et al. (2015) suggested that the distal response might correspond to a combination of biotic and drought/oxidative stress responses.

In this work, we studied both local (woody stem) and distal (leaf) responses to *N. parvum* colonization at the transcriptomic level using RNA sequencing (RNAseq). Genome-wide transcriptional profiling of infected grapevines showed that both organs undergo extensive transcriptomic reprogramming at different time points after inoculation, suggesting a delayed induction of responses in the leaves. Network co-expression analysis identified sets of co-expressed genes common to wood and leaf tissues and showed that many of these genes were asynchronously modulated. The asynchronicity affected the transcriptional modulation of genes involved in both signal perception and transduction, as well as downstream biological processes.

## Materials and methods

### Biological material

**Figure 1:**
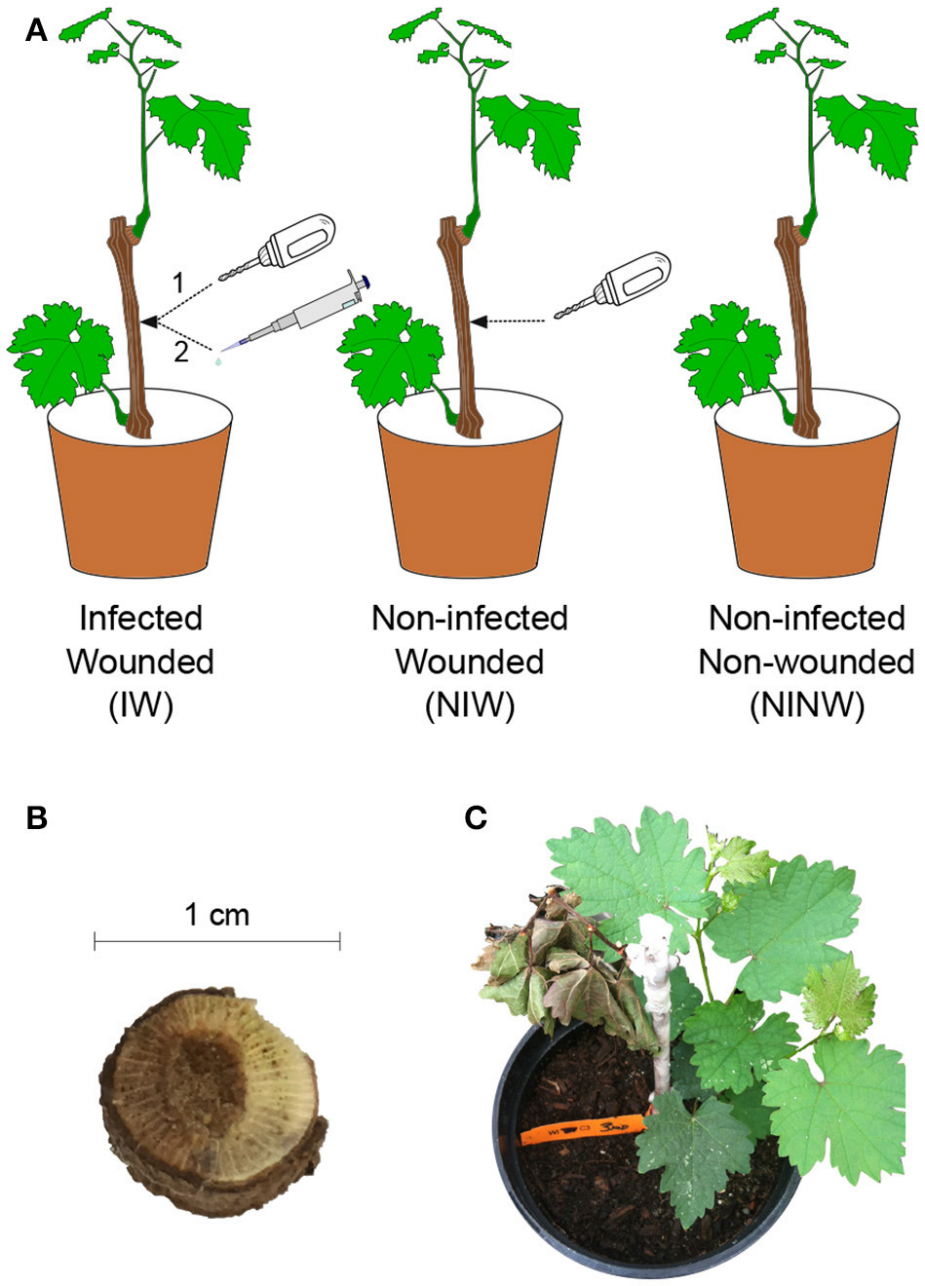
**(A)** Graphical representation of the experimental design and **(B,C)** symptoms of Botryosphaeria dieback observed during the experiment. IW plants are ‘Cabernet sauvignon’ plants that were first wounded using a drill (1) and then inoculated with *N. parvum* isolate UCD646So (2); NIW plants were wounded and mock-inoculated; NINW plants were neither wounded nor infected. **(B)** Grapevine wood canker just below the inoculation site and **(C)** dieback of distal shoot 2 weeks post-inoculation.

**Figure 2:**
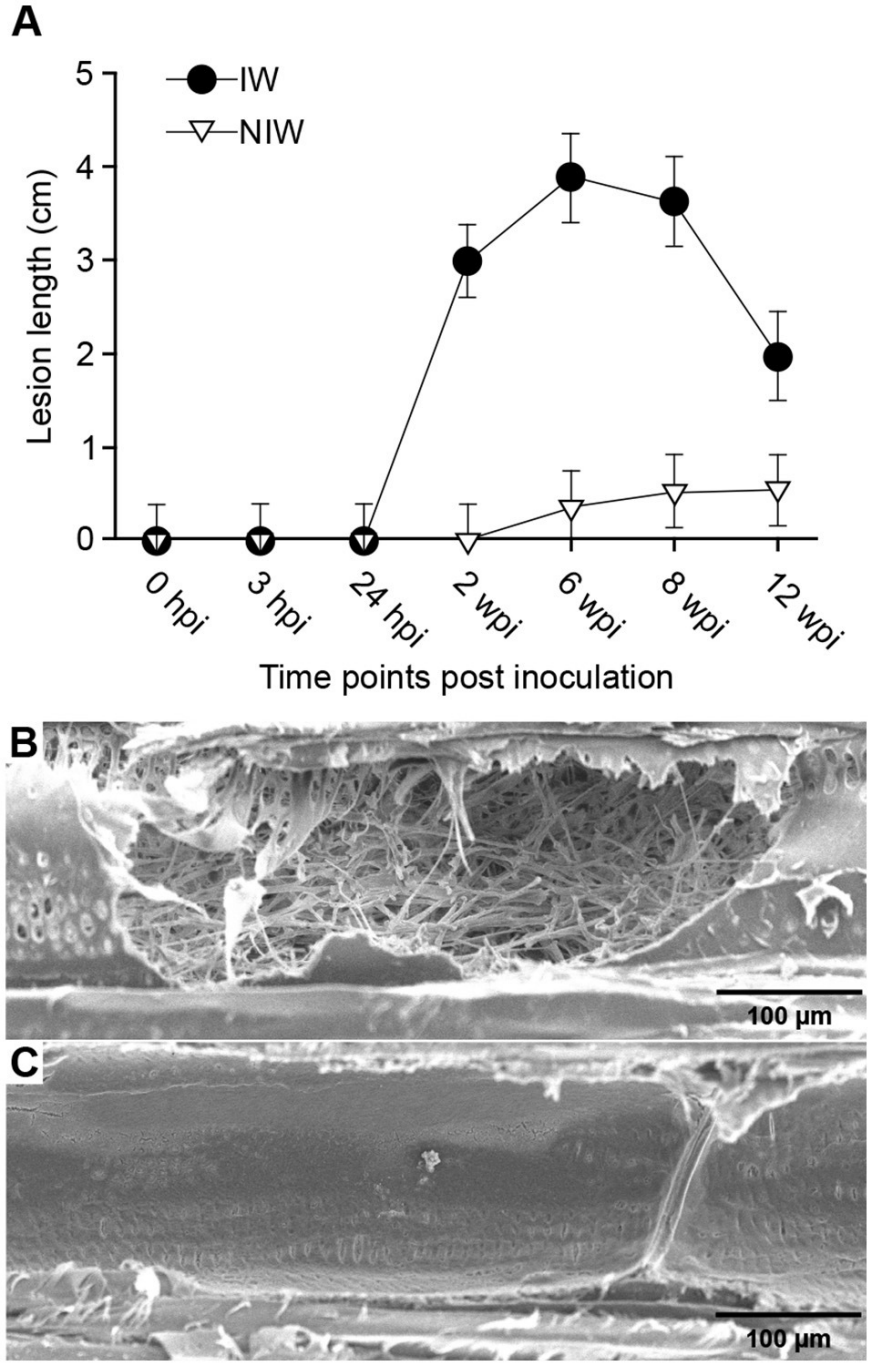
Lesion development and pathogen localization in *N. parvum* infected woody stems. **(A)** Pattern of lesion development during the 12-week time course. Error bars are 95% confidence limits. Points with overlapping error bars are not significantly different (Tukey *post-hoc* test, *P* < 0.05, α = 0.05). NINW plants were not included in the ANOVA because lesion lengths were equal to 0 at all sampling time points. SEM micrographs of tangential longitudinal sections of open secondary xylem vessels of infected plants **(B)** and wounded and non-inoculated plants **(C)**. Panel B shows a vessel lumen extensively colonized by *N. parvum* hyphae and panel C shows an empty vessel lumen with a thin coating of gels on the lateral wall. Bar, 100 μm.

The experiment was conducted on rooted *Vitis vinifera* cv. “Cabernet Sauvignon” clone 19 1-year-old dormant cuttings, propagated in 10 × 10-cm pots 2 months before inoculation, as described by Travadon et al. (2013). In total, 252 plants were arranged in a completely randomized design in a greenhouse [natural sunlight photoperiod, 25 ± 1°C (day), 18 ± 3°C (night)]. Grapevines were subjected to three different treatments as illustrated in Figure 1A. A power drill was used to wound the woody stem (2 × 3 mm) 1 cm below the uppermost node. After wounding, plants were either inoculated with 20 μl of homogenized mycelial fragments of *N. parvum* UCD646So [Inoculated-Wounded plants (IW)] or “mock” inoculated with 20 μl of sterile potato dextrose broth [Non-inoculated Wounded plants (NIW)], then the wound was sealed with Vaseline (Unilever, Greenwich, CT) and Parafilm (American National Can, Chicago, IL) (Travadon et al., 2013). Another set of control plants was neither wounded nor inoculated [Non-inoculated Non-wounded plants (NINW)]. Leaves and woody stems were collected for all three treatments at seven time points: 0, 3, and 24 h post-inoculation (hpi), and 2, 6, 8, and 12 weeks post-inoculation (wpi). Time points were selected based on previous observations on the pathosystem, which indicated that plant responses to *N. parvum* infection are activated weeks post-inoculation (Czemmel et al., 2015). At each time point, the two youngest leaves from each plant that were at least 2-cm in width and pieces of wood spanning 2 cm above and below the inoculation site were sampled using flame-sterilized clippers and forceps, immediately placed in liquid nitrogen, and subsequently stored at −80°C for later RNA extraction. Sampling at 0 hpi was done within 10 min of inoculation. Infections were confirmed by positive culture-based recovery of the pathogen after 5-day growth on potato dextrose agar, and internal lesion lengths were measured at each sampling time point (Figure 2A; Data S1). Steps for recovery were processed as described in Czemmel et al. (2015). For each treatment and time point, three biological replicates were collected. Each biological replicate corresponded to an individual plant for stem samples. For leaf samples, each biological replicate consisted of a pool of four plants to obtain enough RNA for subsequent analyses. Separate sets of plants were used for RNA sampling, for lesion measurement and pathogen recovery, and for microscopy.

### Microscopy

Stem xylem tissues were analyzed by conventional scanning electron microscopy (SEM) following the protocol of Sun et al. (2011). Briefly, stem samples were collected and immediately fixed in a formalin glutaraldehyde solution for over 48 h. Then, 1 mm thick xylem segments were cut from 2 cm below the inoculation site of each fixed sample, exposing transverse or longitudinal surfaces. The trimmed xylem segments were dehydrated through an ethanol series, critical-point-dried in a Denton Vacuum DCP-Critical Point Dryer (Denton Vacuum LLC, USA), coated with gold and palladium in a Denton Vacuum Desk II Sputter Coater (Denton Vacuum, Inc., Moorestown, NJ, USA) and finally observed under a SEM (Hitachi S3400N, Hitachi Science Systems, Ltd., Tokyo, Japan) at an accelerating voltage of 8 kV.

### RNA extraction and library preparation

Three biological replicates, corresponding to a pool of leaves from four plants for the distal sampling, and to individual stems (−1 cm) for the local sampling, were used for RNA extraction. Total RNA was isolated using a CTAB-based extraction protocol followed by a DNase treatment as described in Blanco-Ulate et al. (2013b). RNA concentration and purity were measured using a Quibit fluorometer (Thermo Scientific) and a NanoDrop 2000c Spectrophotometer (Thermo Scientific), respectively. Libraries were prepared using the Illumina TruSeq RNA sample preparation kit v.2 (Illumina, CA, USA). Final libraries were evaluated for quantity and quality using the High Sensitivity DNA kit on a Bioanalyzer 2100 (Agilent Technologies, CA).

### RNA sequencing and downstream analyses

cDNA libraries were sequenced using an Illumina HiSeq3000 sequencer (DNA Technologies Core, University of California, Davis, California, USA) as single-ended reads of 50 nucleotides (Illumina, CA, USA). Raw reads were deposited to the National Center for Biotechnology Information's Gene Expression Omnibus (GEO) and are accessible through GEO (GSE97900 accession). Due to much greater coverage compared with the other samples, the third replicate of the 8 wpi-NINW sample was reduced to 17 M reads by random read sampling using fastq-sample (v0.8; https://github.com/dcjones/fastq-tools). Quality trimming (*Q* > 20) and adapter contamination removal were carried out using sickle (v1.2.1; Joshi and Fass, 2011) and scythe (v0.991; Buffalo, 2011), respectively. The predicted transcriptome of *N. parvum* UCD646So was combined with the predicted transcriptome of *V. vinifera* cv. “PN40024” (version V1 from http://genomes.cribi.unipd.it/grape/) and used as a reference for mapping stem- and leaf-sample reads. Bowtie2 (v2.2.327; Langmead and Salzberg, 2012) was used to align the quality-trimmed reads to the combined references with parameters: -q -end-to-end -sensitive -no-unal -p 20. Mapping counts were extracted using sam2counts.py (v0.91 https://github.com/vsbuffalo/sam2counts). Details on results of data trimming and mapping are reported in Table S1. The Bioconductor package DESeq2 (Love et al., 2014) was used to perform read count normalization of the grape genes and differential expression (DE) analysis across treatments (i.e., IW vs. NIW, IW vs. NINW, NIW vs. NINW) at each time point. Genes were considered differentially expressed (DEGs; adj. *P* ≤ 0.05) in response to *N. parvum* infection if they were significantly up- or down-regulated in IW samples in comparison to control samples (IW vs. NINW). From this set we excluded genes that were DE in response to both wounding (NIW) and *N. parvum* (IW) when compared to NINW, but not DE when pathogen infected (IW) samples are compared with wounded samples (NIW). This additional filtering was carried out to remove genes apparently modulated by wounding also in presence of the pathogen. Relative overlap of DEGs in local and distal datasets was calculated for each pairwise comparison by dividing the number of genes sharing the same modulation (up- or down-regulated) in both leaf and stem samples by the total (non-redundant) number of DEGs in that specific comparison. Up-regulated and down-regulated genes were processed separately. Overlap values were converted in distances by subtracting the overlap values to 1 and converted to a dendogram by hierarchical clustering using heatmap 0.2 function from the gplots R package (Warnes et al., 2009).

### Weighted gene co-expression network analysis

Genes that were identified as DEG in response to *N. parvum* infection in both tissues were the input for the Weighted Gene Co-expression Network Analysis (WGCNA). Prior to statistical testing, we added “one” to each normalized count value, calculated the log_2_ fold-change values between IW and NINW samples and converted to “zero” all fold-change values associated with comparisons that were not considered significant by DESeq2 as described in Amrine K. C. et al. (2015). A gene co-expression network was constructed from each tissue sample dataset using the blockwiseModules R function in WGCNA v.1.51 (Langfelder and Horvath, 2008). The default soft-thresholding power of 12 for signed networks was used (Horvath, 2011), with a scale-free model fitting index *R*^2^ > 0.52 for the stem network and *R*^2^ > 0.08 for the leaf network (Figure S1). A relatively large minimum module size of 30, a medium sensitivity (deepSplit = 2) to cluster splitting, and a threshold of 0.25 for the merging of modules were used. Low values of the scale-free model fitting index might be either due to the filtering of the genes by differential expression, or caused by the sample heterogeneity observed for each tissue (Figure S2). Despite the low fit to a scale-free topology, the validity of the WGCNA approach was supported by the structure of both dendograms where modules belonged to distinct branches. In each network, module eigengenes were extracted (Data S2) and averaged by time point. A module-time point relationship matrix was generated for each network in order to confirm the correlation between module eigengene and time points (Figure S3; Langfelder and Horvath, 2014). Significance of each module overlap between stem and leaf networks was computed using the Fisher exact test, and a cutoff of *P* ≤ 0.01 was set to determine statistical significance. The overall significance of module preservation was assessed using Z_summary_ that combines multiple preservation statistics into a single overall measure of preservation as described by Langfelder et al. (2011) (Figure S4). For network topological analyses, stem and leaf unweighted networks were extracted using a hard threshold of TOM > 0.1 and imported into Cytoscape v3.4 (Shannon et al., 2003). For each gene, the node degree was extracted. Grapevine promoter sequences (i.e., 1 and 5 kb upstream of the coding sequence) of genes belonging to the 5 largest module overlaps were retrieved from Gramene v52 (http://www.gramene.org/) based on the 12x grapevine genome assembly. For each module overlap, available sequences were analyzed using the MEME SUITE (Bailey et al., 2009). MEME v4.11.1 was run with the following parameters: -dna -mod zoops -nmotifs 20 -evt 0.01 with a motif width from 8 to 21 k-mers, according to the width distribution of the known plant promoter motifs among the PlantTFDB v4.0 database (Jin et al., 2017). Motifs were annotated by comparison with PlantTFDB v4.0 database (Jin et al., 2017) using tomtom v4.11.1 with the following parameters: -e-value -no-ssc. The VitisNet functional annotations were used to assign grape genes to functional categories (Grimplet et al., 2009). Enrichment analyses of grape biological functions were computed in R using the classic Fisher method (*P* ≤ 0.01).

### RT-qPCR

The relative expression of 5 genes, one gene for each of the 5 largest module overlap, at 24 hpi and 2 wpi in both stem and leaf tissues, was used to validate the RNAseq data. First, cDNA was prepared from total RNA using Moloney murine leukemia virus reverse transcriptase (Promega, Madison, WI, USA). Then, quantitative PCR (qPCR) was performed on a QuantStudio 3 Real-Time PCR System (Thermo Fisher Scientific, Waltham, MA, USA) using the Power SYBR Green Master Mix (Applied Biosystems, Foster City, CA, USA). The qPCR conditions used to amplify all genes were as follows: 50°C for 2 min, 95°C for 10 min, followed by 40 cycles of 95°C for 15 s and 60°C for 1 min. *VvACTIN* (*VIT_04s0044g00580*) and Ubiquitin-like (UBX)-domain-containing protein gene (*VIT_05s0029g01370*) were selected as reference genes. *VvACTIN* was selected because of its expression stability in grapevine tissue during biotic stress (Amrine K. C. H. et al., 2015; Blanco-Ulate et al., 2015, 2017). The gene encoding a UBX-domain-containing protein was identified in this study as a constitutively expressed gene in both stem and leaf tissues based on its low coefficient of variation (6%) across all biological triplicates. Three biological replicates of each tissue (stem and leaf) and condition (IW and NINW) were used to obtain the relative gene expression data. Primer sequences were designed spanning multiple exons using Primer3 v0.4.0 (Untergasser et al., 2012; Table S2). Primer efficiency was calculated using 4-fold cDNA dilutions (1:1, 1:4, 1:16, 1:64, and 1:256) in triplicate, while primer specificity was checked by analyzing the dissociation curves at temperatures ranging from 60 to 95°C (Table S2). In addition, genomic DNA contamination was ruled out by performing a qPCR assay using total RNAs as a template. Fold changes in gene expression were calculated using the 2^−ΔΔCt^ method (Livak and Schmittgen, 2001). Relative changes in gene expression obtained with the UBX-domain-containing protein gene as reference gene provided a similar correlation to *VvACTIN* (*R* = 0.94; *P* < 10^−12^; Figure S5), which makes it suitable for RT-qPCR validation of gene expression in this experiment.

### Chemical analysis

For chemical analysis, grapevine stems were collected and immediately frozen in liquid nitrogen as described above. Stem pieces above and below the inoculation site (+1 and −1 cm, respectively) for three samples per treatment × time point were pooled and ground into a powder in liquid nitrogen using a TissueLyser II (Qiagen, Hilden, Germany) with stainless steel grinding jars. Phenolics were extracted using methanol and then analyzed using High-Throughput Liquid Chromatography (HPLC) as described in Wallis and Chen (2012), as well as peaks identification and compounds quantification (Table S3). In particular cases, when no peak was detected for a certain compound, compound was set to 1,000 AUs before converting to gram amounts. Concentrations of compounds in the same phenolic class, such as stilbenoids, catechins, and procyanidins, were summed together for statistical analyses. Concentrations were then log_2_-transformed and analyzed statistically at each time point by one-way analysis of variance (ANOVA), followed by Tukey's HSD test using the statistical R package agricolae (*P* < 0.05; De Mendiburu, 2009).

## Results

### *Neofusicoccum parvum* colonization of grapevine woody stems

Symptoms of Botryosphaeria dieback, such as internal necrotic wood cankers and the death of distal shoots, were observed throughout the duration of the experiment, starting at 2 wpi (Figures 1B,C, respectively). *N. parvum* was recovered from the inoculation site (0 cm) of all stems at all timepoints for the IW plants, while it was recovered beyond the inoculation site (1 cm above and below) starting at 2 wpi (Data S1 Table A). As expected, the pathogen was not recovered in culture from stem samples of NIW and NINW plants at any time point. Lesions due to *N. parvum* infection were detectable only after 2 wpi, and reached their largest lengths by 6 and 8 wpi (Figure 2A; Data S1 Table B). The apparent decline of mean lesion size observed at 12 wpi is likely due to mortality of IW plants, which were removed from the study, between 8 and 12 wpi. Additional observations of stem sections during *N. parvum* infection using conventional (SEM) showed in IW plants the extensive colonization of vessel lumens by fungal hyphae (Figures 2B,C).

### Profiling of woody stem and leaf transcriptomes during *N. parvum* colonization

**Figure 3:**
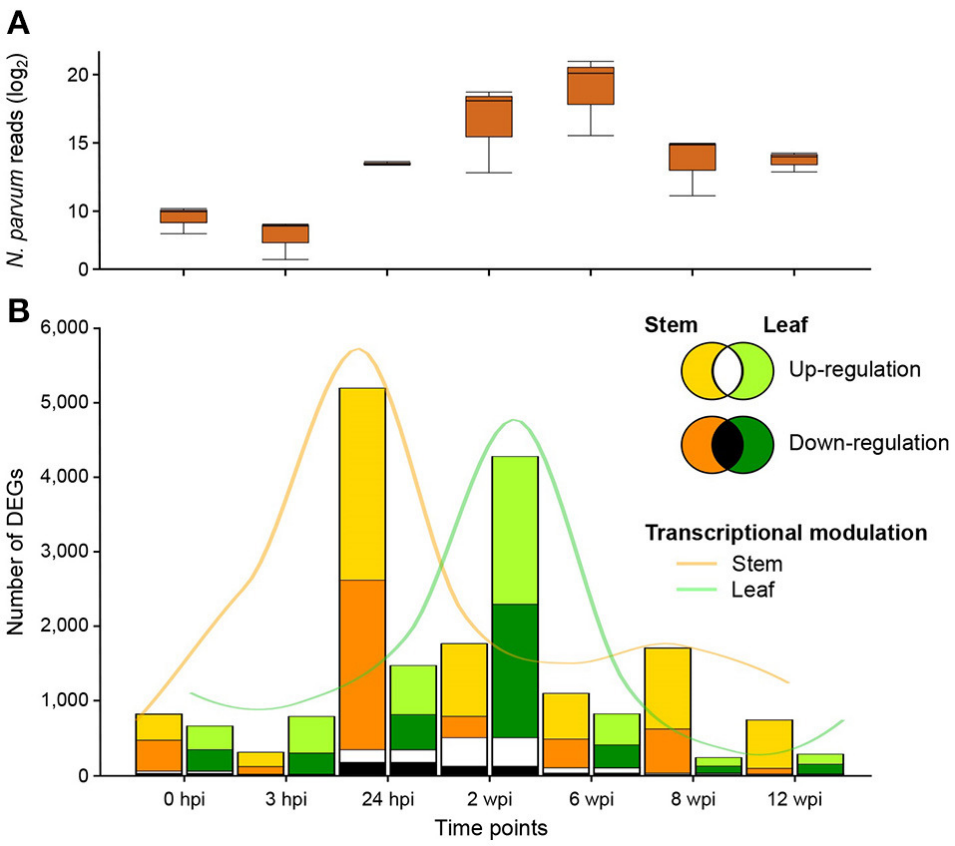
Differentially expressed genes (DEGs) during *N. parvum* infection. **(A)** Number of reads (log_2_) mapped on *N. parvum* transcriptome during the time course of fungal colonization. **(B)** Number of grape genes up- and down-regulated in stem (gold and orange, respectively) and leaf (light and dark green, respectively) tissues during *N. parvum* infection. Number of genes commonly up- and down-regulated in the two organs at each time point are represented in white and black color, respectively.

To characterize the responses to *N. parvum* in the woody stem at the site of inoculation (local response) and in the leaves (distal response), we profiled the stem and leaf transcriptomes using RNAseq at the seven time points described above. For sequencing read mapping, we used a transcriptome reference that comprised all predicted transcripts of *N. parvum* UCD646So (Massonnet et al., 2016) and grape (*V. vinifera* var. “PN40024”; Jaillon et al., 2007). An average of 13 ± 2.7 million quality-trimmed reads were aligned to the combined reference (Table S1). As described previously (Massonnet et al., 2016), the number of reads derived from *N. parvum* transcripts in woody stems was low at 0 and 3 hpi (830 ± 362 and 367 ± 198, respectively), increased from 24 hpi (11,439 ± 900) to 6 wpi (1,071,368 ± 816,298), and declined at 8 and 12 wpi (14,423 ± 5,087) (Figure 3A). This pattern likely reflected the accumulation of fungal biomass from 24 hpi to 6 wpi, followed by a progressive decline possibly due to further colonization of woody tissue beyond the inoculation site. The transcriptomic dynamics of *N. parvum* during colonization of woody stems was described in depth in Massonnet et al. (2016). In leaves, only an average of 6 ± 2.9 reads per sample across the three treatments aligned to the *N. parvum* transcriptome. The negligible number of reads assigned to the fungal transcriptome confirmed that *N. parvum* did not colonize leaf tissues.

Grape transcript counts were normalized and differential expression analysis across treatment combinations (Table S4) was performed at each time point using DESeq2 (Love et al., 2014). The wound treatment alone (NIW vs. NINW) induced significant changes in the transcriptome of both tissues over the experiment time course [302 ± 288 and 546 ± 429 differentially expressed genes (DEGs) at each time point in stem and leaf, respectively]. However, infection by *N. parvum* (IW vs. NINW) led to the differential regulation of a larger number of genes (1,798 ± 1,622 and 1,545 ± 1,412 DEGs in stem and leaf, respectively). DEGs in response to either wounding or infection were then compared to identify genes whose expression was significantly altered in response to infection at each time point (see Material and Methods; Data S3). A total of 10,810 DEGs were detected in response to infection across the seven time points. A greater number of genes was differentially regulated in the stems (7,874 DEGs) than in leaves (5,914 DEGs). The most extensive transcriptional reprogramming caused by infection was observed in stems at 24 hpi (5,196 DEGs) and at the next time point (2 wpi) in leaves (4,279 DEGs; Figure 3B). These results suggested that both tissues undergo major transcriptional reprogramming in response to *N. parvum*, but at different time points.

### At each time point, infection induces different responses in stems and leaves

**Figure 4:**
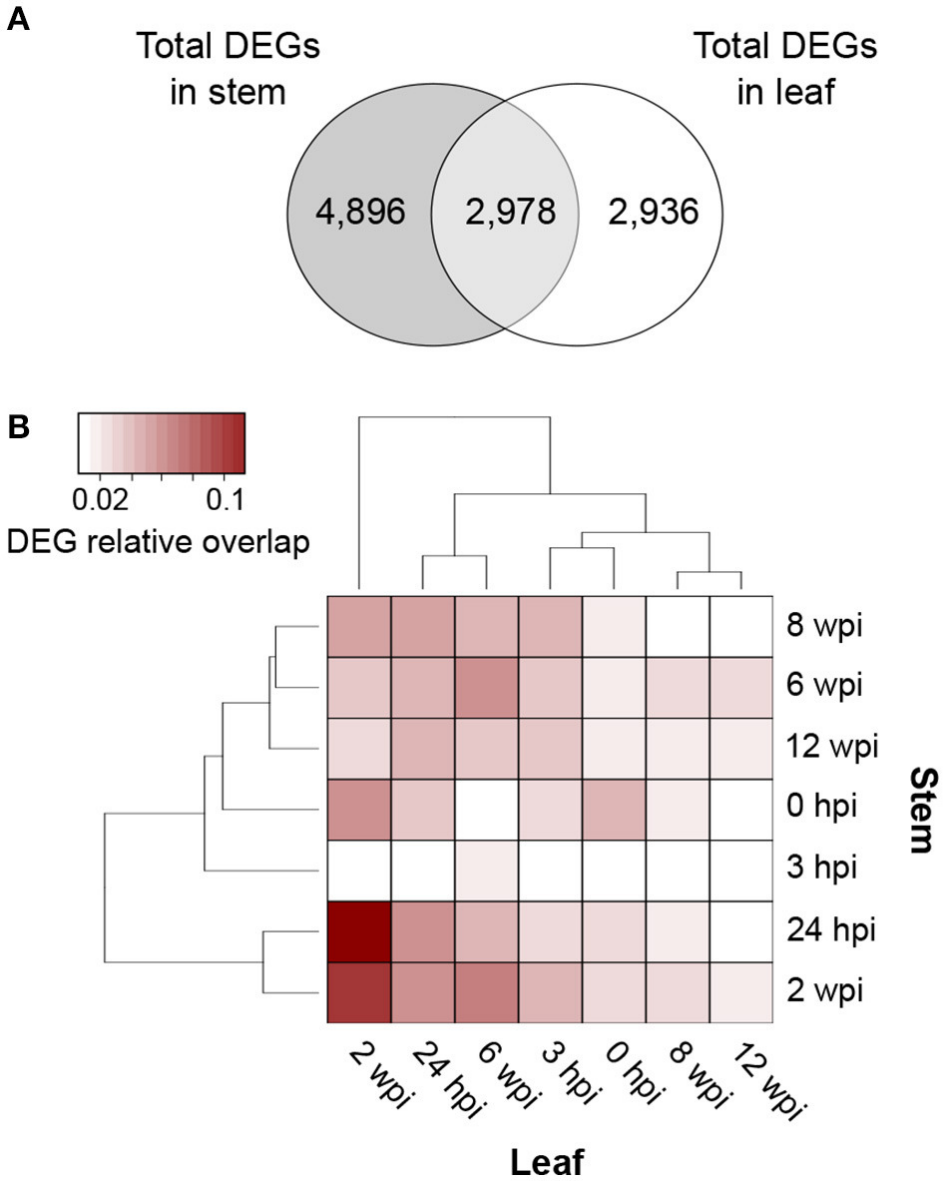
Comparisons of differentially expressed genes (DEGs) in response to *N. parvum* infection. **(A)** Venn diagram displaying common and unique DEGs (adjusted *P* ≤ 0.05) when comparing responses to *N. parvum* colonization in stem and leaf tissues. **(B)** Heatmap of the relative DEG overlap between stem and leaf tissues during *N. parvum* infection.

**Figure 5:**
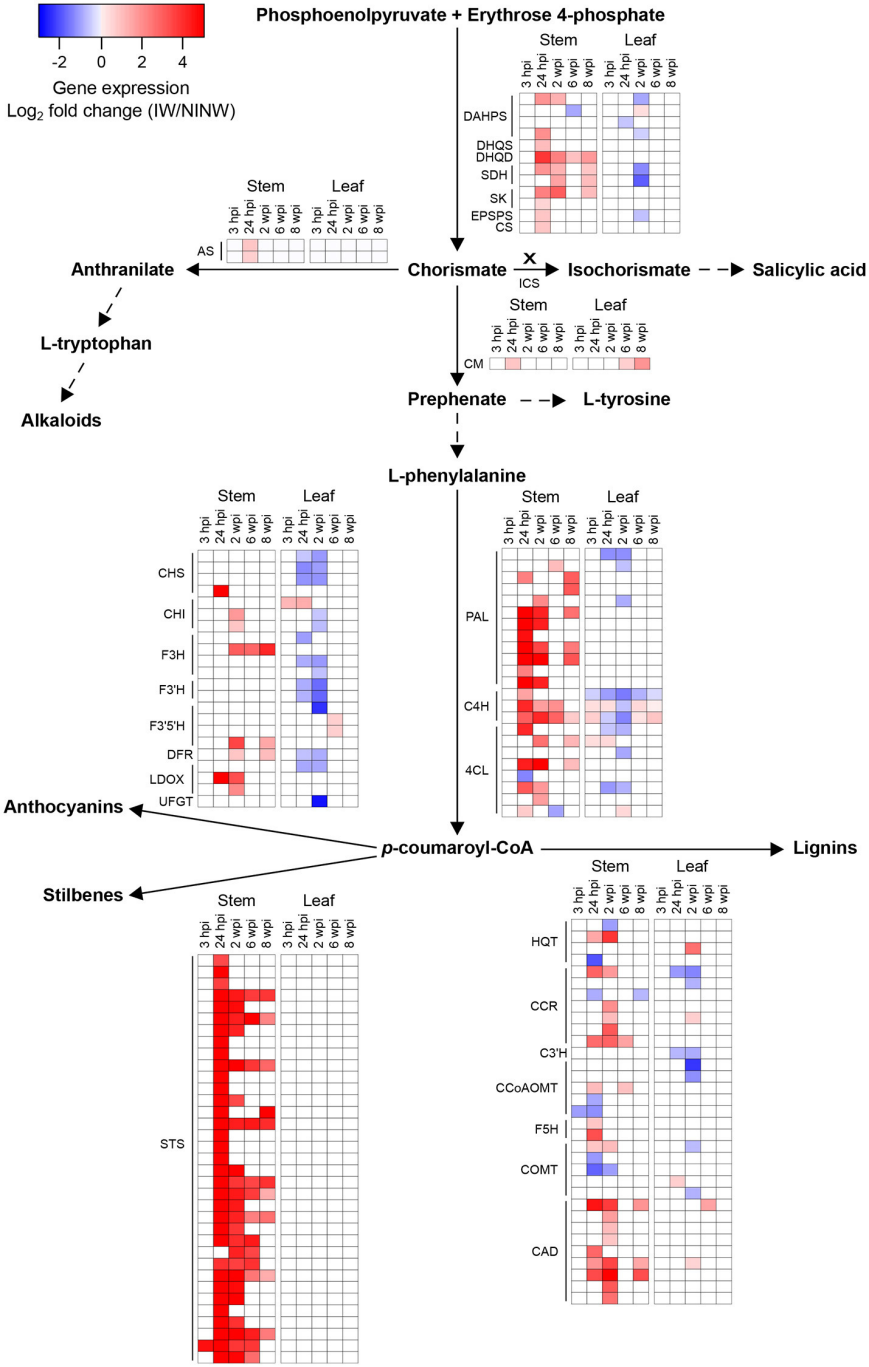
Transcriptional modulation of the secondary metabolism-associated genes in response to *N. parvum* infection. Representation of the shikimate, phenylalanine, central phenylpropanoid, anthocyanin, stilbene and lignin biosynthetic pathways based on Kyoto Encyclopedia of Genes and Genomes pathways (www.genome.jp/kegg/pathway.html) is provided. Dashed lines indicate that the gene expression modulation is not represented for the corresponding biosynthetic pathway. The cross above the arrow indicates that no transcriptional modulation of ICS genes has been identified. Heat maps depict significant fold changes (log_2_) in expression of fungal infection-responsive genes, coding for biosynthetic enzymes involved in each pathway, in stem and leaf tissues from 3 hpi until 8 wpi. Colors in the heat maps represent the intensity of the expression changes. Shikimate biosynthetic pathway: DAHPS, 3-deoxy-D-arabino-heptulosonate 7-phosphate synthase; DHQS, 3-dehydroquinate synthase; DHQD, Dehydroquinate dehydratase; SDH, Shikimate dehydrogenase; SK, Shikimate kinase; EPSPS, 5-enolpyruvylshikimate 3-phosphate synthase; CS, Chorismate synthase. Phenylalanine biosynthetic pathway: AS, Anthranilate synthase; CM, Chorismate mutase. Salicylic acid biosynthetic pathway: ICS, Isochorismate synthase. Central phenylpropanoid biosynthetic pathway: PAL, Phenylalanine ammonia-lyase; C4H, Cinnamate 4-hydroxylase; 4CL, 4-coumarate-CoA ligase. Anthocaynin biosynthetic pathway: CHS, Chalcone synthase; CHI, Chalcone isomerase; F3H, Flavonone-3-hydroxylase; F3′H, Flavonoid-3'-hydroxylase; F3′5′H, Flavonoid-3',5'-hydroxylase; DFR, Dihydroflavonol 4-reductase; LDOX, Leucoanthocyanidin dioxygenase; UFGT, UDP-glucose:flavonoid 3-O-glucosyltransferase. Stilbene biosynthetic pathway: STS, Stilbene synthase. Lignin biosynthetic pathway: HQT, Hydroxycinnamoyl-CoA quinate hydroxycinnamoyltransferase; CCR, Cinnamoyl-CoA reductase; C3′H, P-coumaroyl 3'-hydroxylase; CCoAOMT, Caffeoyl-CoA O-methyltransferase; F5H, Ferulate 5-hydroxylase, COMT, Caffeic acid *O*-methyltransferase; CAD, Cinnamyl alcohol dehydrogenase.

**Figure 6:**
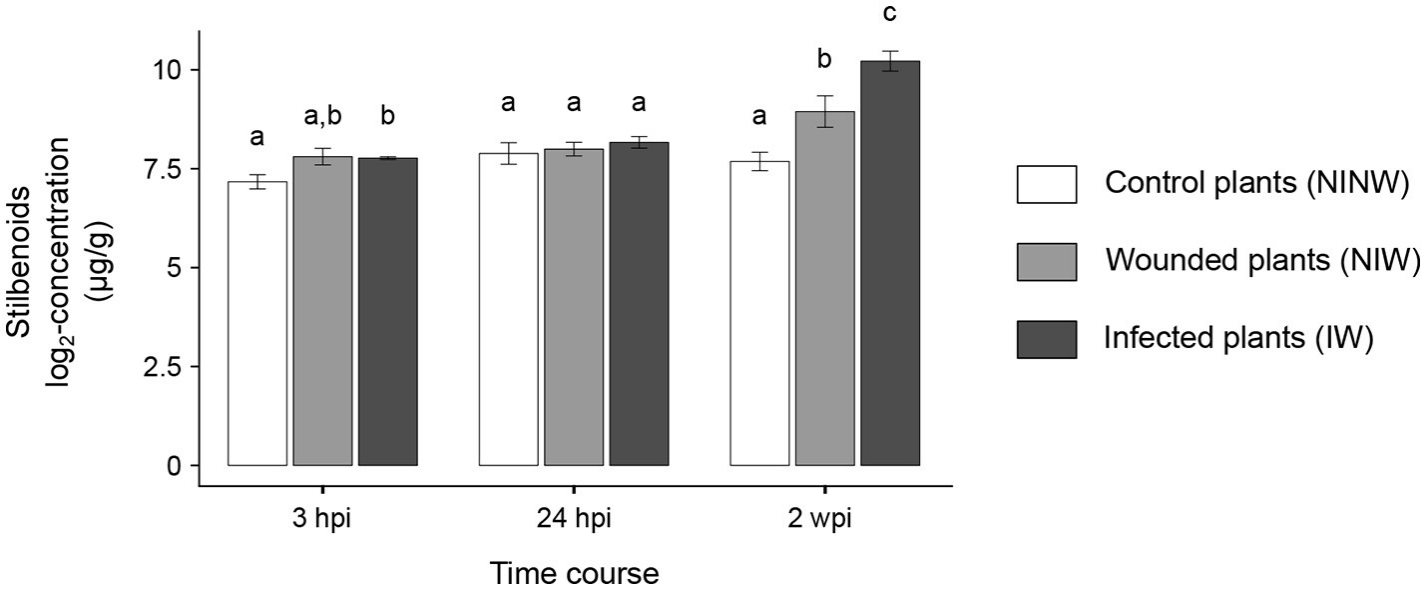
Stilbenoid compounds concentrations in woody stems. Stilbenes data are expressed as mean ± standard deviation. ANOVA followed by Tukey's *post-hoc* test was used to compare the log_2_-transformed concentrations between the three treatment conditions (IW, NIW, NINW) at each time point. Adjusted *P* < 0.05 was considered statistically significant. At each time point, samples with the same letter are not significantly different.

Overall, local and distal responses shared 2,978 DEGs, corresponding to 37.8% and 50.3% of the total number of DEGs in stems and leaves, respectively (Figure 4A; Data S3). However, at each time point, the two organs displayed a limited overlap in the set of DEGs (Figure 3B). The comparison of the enriched functional categories (FCTs; Fisher's exact test, *P* ≤ 0.01) among the up- and down-regulated genes at each time point in both organs (Data S3) showed that the FCTs related to secondary metabolism, such as “Shikimate metabolism,” “Phenylpropanoid biosynthesis,” “Anthocyanin-glycoside biosynthesis,” “Stilbenoid biosynthesis,” and “Lignin metabolism,” were significantly enriched only among up-regulated genes in the stem. Expression of secondary metabolism genes was particularly enriched in the stem at 24 hpi and 2 wpi. In the leaves at these time points, “Shikimate metabolism” and “Anthocyanin biosynthesis” were overrepresented among the down-regulated genes (Data S3). Infection induced the up-regulation of 38 stilbene synthase (STS)-encoding genes in the stem at 24 hpi (Figure 5), while no *STS* gene was differentially expressed in leaves. Indeed, the concentration of stilbenes significantly increased at 2 wpi in the stems, likely reflecting the up-regulation of *STS* genes at 24 hpi (Figure 6). No significant change in catechin and procyanidin concentration was detected (Figure S6). Several genes involved in lignin biosynthetic process as well as 39 laccase-coding genes were exclusively up-regulated in the stem (Figure S7). The FCT “Citric acid cycle” was significantly enriched among the genes up-regulated in stems at 24 hpi and 2 wpi, including 15 genes whose up-regulation was specific to wood (Figure S8). The stem response also involved the induction of a larger number of reactive oxygen species (ROS) scavenging-associated genes and PR protein-encoding genes not seen in the distal response (Figures S9–S11). A similar pattern of differential regulation between stems and leaves was found for genes associated with hormone-mediated signaling pathways. For example, the *MYC2* gene (*VIT_02s0012g01320*), a regulator of jasmonic acid signaling was found up-regulated exclusively in stems. We also observed the specific up-regulation of the putative negative regulator of systemic acquired resistance (SAR) *VvNPR1.2* (*VIT_10s0042g01250*) in leaves starting at 24 hpi until 6 wpi (Bergeault et al., 2010; Le Henanff et al., 2011).

### Local and distal responses involved the asynchronous transcriptional modulation of a common set of co-expressed genes

Due to the peak of differential regulation at 24 hpi and 2 wpi in stem and leaves, respectively, we analyzed the RNAseq data to determine if, despite that limited overlap of DEGs at each time point, the two organs activated similar responses asynchronously. Pairwise comparisons of all stem and leaf samples identified time points at which stem and leaves share DEGs (Figure 4B). The largest relative overlap of DEGs [10.0% (461 DEGs) up- and 12.9% (500 DEGs) down-regulated genes] was found between the stem and leaf transcriptomes at 24 hpi and 2 wpi, respectively. In addition to sharing a set of common DEGs, stems at 24 hpi and leaves at 2 wpi of IW plants also showed similar enriched FCTs (Fisher's exact test, *P* ≤ 0.01) among their corresponding DEGs (Data S3). Common functions in the up-regulated DEGs included “Protein kinase,” “Ethylene (ET)-,” “Jasmonate (JA)-,” and “Salicylic acid (SA)-mediated signaling pathways,” “WRKY family transcription factor,” and “Cell death”; “Regulation of cell cycle,” “Microtubule-drive movement,” and “AUX/IAA family transcription factor” were identified as significantly enriched among the down-regulated genes in both tissues. These results suggest that the two organs activated common responses asynchronously upon infection.

**Figure 7:**
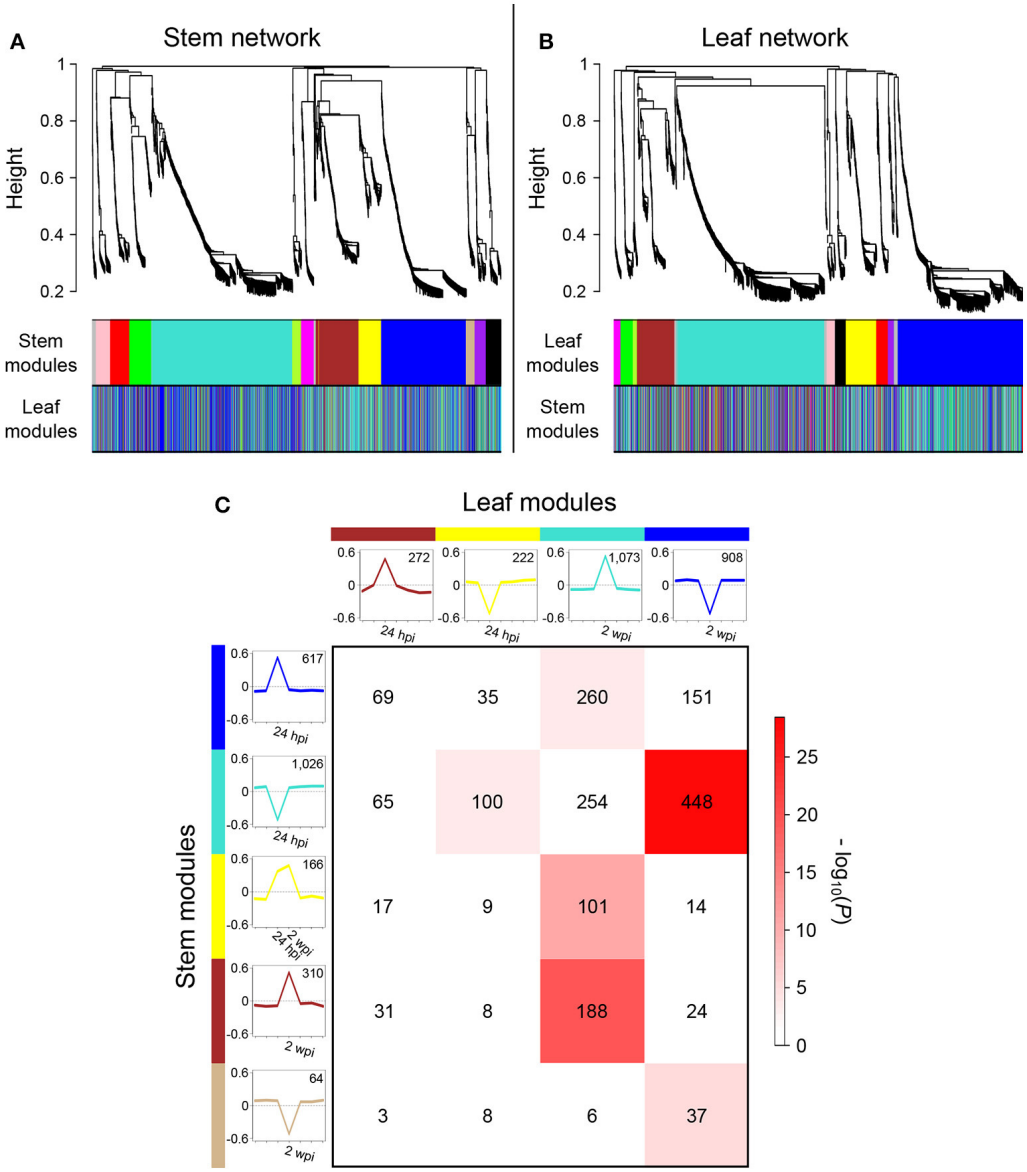
Comparison of stem and leaf co-expression networks. **(A)** Hierarchical clustering tree (dendrogram) of genes based on stem network. Each short vertical line corresponds to one gene. The colored rows below the dendrogram indicate module membership in the stem modules and in the leaf network. **(B)** Hierarchical clustering tree of genes based on the leaf network. The color rows below the dendrogram indicate module membership in the leaf modules and in the stem network. **(C)** Cross-tabulation of stem (rows) and leaf modules (columns) showing a transcriptional modulation at 24 hpi and 2 wpi. Each row and column is represented by the corresponding module color. In the table, numbers give counts of genes in the intersection of the corresponding row and column module. The table is color-coded by -log*(P*), the Fisher exact test *P*-value, according to the color legend on the right. For each module, the eigengene expression profile is color-coded with the corresponding module color and the module size is indicated. The eigengene can be interpreted as a weighted average gene expression profile (Langfelder et al., 2011).

To gain further insights into the asynchronous transcriptional responses triggered by infection and to determine whether patterns of co-expression are shared between local and distal responses, suggesting that common regulatory mechanisms are involved, we constructed and compared gene co-expression networks of stems and leaves. Co-expression network analysis was carried out using WGCNA (Langfelder and Horvath, 2008) for each organ, based on the 2,978 DEGs common in both organs. We identified 12 modules of co-expressed genes in the stem network (Figure 7A) and 11 in the leaf network (Figure 7B; Data S2). Following the WGCNA pipeline, a unique color label was assigned as a specific module identifier. For each network, module eigengenes were calculated to obtain the most representative gene expression profile for each co-expression module (Figures S12A,B). This allowed the visualization of gene expression profile trends in the two co-expression networks, which showed that the two largest modules of both networks (“blue” for stem, “turquoise” for leaves), contained co-expressed genes modulated at 24 hpi in stems and 2 wpi in leaves (Figure 7C; Figure S12). To identify co-expressed genes in common between leaf and stem modules, a contingency table was generated using a cross-tabulation approach (Figure S12C). The analysis detected 12 significant module overlaps (*P* < 0.01) between the two networks, corresponding to a total of 1,283 genes (43% of the common DEGs). Composite preservation statistics identified the “turquoise,” “blue,” and “brown” modules of the stems as moderately preserved in leaves, as well as the leaf “blue” module conserved in the stem dataset (Figure S4). The largest overlap was between the stem “turquoise” and leaf “blue” modules corresponding to 448 genes (43.7 and 49.3% of each module size, respectively), which exhibited a down-regulation at 24 hpi in stems and at 2 wpi in leaves (Figure 7C). The DEGs in common between the two organs were significantly enriched in the FCTs “Cell growth and death,” “Regulation of the cell cycle,” “Microtubule-driven movement,” “Chromatin assembly,” and “Cell wall organization and biogenesis” (Table 1). The second largest overlap was between the stem “blue” and leaf “turquoise” modules. The two modules shared 260 genes with a peak of expression at 24 hpi in stems and 2 wpi in leaves. These 260 genes were significantly enriched in the FCTs “Plant-pathogen interaction” including eight resistance protein (R) genes, the “SA-mediated signaling pathway” with the Phytoalexin-deficient (PAD) 4 protein gene *VvPAD4* (*VIT_07s0031g02390*) and the two Enhanced disease susceptibility 1 (EDS1)-like protein genes *VvEDL1* and *VvEDL5* (*VIT_17s0000g07370* and *VIT_17s0000g07420*, respectively), the “Calcium sensors and signaling,” and the “Protein kinase” with 29 genes including 14 serine/threonine kinase genes and four wall-associated kinases likely involved in plant defense (Afzal et al., 2008; Delteil et al., 2016). The “WRKY family transcription factor” FCT was also significantly overrepresented in this set of genes and included *VvWRKY2* (*VIT_01s0011g00220*), which was shown to be involved in grape resistance against necrotrophic fungal pathogens and in regulation of lignin deposition (Mzid et al., 2007; Guillaumie et al., 2010). One hundred co-expressed genes up-regulated from 24 hpi to 2 wpi in stems (stem “yellow” module) were also co-expressed in leaves with an up-regulation at 2 wpi (leaf “turquoise” module). In this set of genes, the FCTs “Cell death,” “ET-mediated signaling pathway,” and “Protein kinase” were found as significantly overrepresented. Among the genes associated with the “ET-mediated signaling pathway” functional category were four Ethylene Responsive Factor/APETALA2 (ERF/AP2) transcription factor genes, as well the mitogen-activated protein kinase (MPK) gene *VvMPK12* (*VIT_06s0004g03540*). *VvMPK12* is phylogenetically close to *AtMPK3* (Çakır and Kılıçkaya, 2015), which was shown to be activated in response to pathogens and abiotic stresses (Colcombet and Hirt, 2008) and to participate in the regulation of the biosynthesis of ET (Xu et al., 2008). These three module overlaps suggested that local and distal responses involved the co-expression of a common set of genes, but also that the modulation of most of these genes was delayed in leaves in comparison to stems.

**Table 1:** Enriched functional categories (*P* ≤ 0.01) in the five largest overlaps between modules of co-expressed genes in stem and leaves during *N. parvum* infection.

**Module overlap**	**Modulation**		**Functional category**	**Gene number**			***P*-value**
**Stem-Leaf (n°)**	**Stem**	**Leaf**		**Stem**	**Leaf**	**Overlap**	
Turquoise-Yellow (100)	↓ 24 hpi	↓ 24 hpi	Photosynthesis-Antenna proteins	8	6	6	4.42E-11
			Photosynthesis-Reaction center pigment biosynthesis	3	3	2	5.94E-04
			Plant photosystem I supercomplex	7	7	6	8.69E-09
			Chlorophyll biosynthesis	3	3	2	1.12E-03
			Thylakoid targeting pathway	9	5	5	7.77E-07
			HSP-mediated protein folding	29	18	11	2.45E-12
			Protein processing in endoplasmic reticulum	21	10	6	1.03E-04
			Circadian clock signaling	5	3	3	2.46E-03
			Nudix hydrolase family	2	2	2	3.66E-03
			Inward rectifier K+ channel	2	1	1	9.98E-03
Brown-Turquoise (188)	↑ 2 wpi	↑ 2 wpi	Xyloglucan modification	15	13	9	5.82E-12
			Peroxisome organization and biogenesis	7	9	6	6.75E-06
			Glyoxylate and dicarboxylate metabolism	5	11	4	1.35E-03
			Glutathione metabolism	6	13	5	2.06E-03
			Cytochrome P450 oxidoreductase	7	13	6	9.89E-03
			Virus stress response	2	2	2	6.24E-03
			ERF subfamily transcription factor	3	6	3	5.28E-03
			Carbon fixation	6	9	4	5.68E-03
Blue-Turquoise (260)	↑ 24 hpi	↑ 2 wpi	Protein kinase	53	85	31	7.26E-06
			Calcium sensors and signaling	17	25	13	2.19E-05
			Salicylic acid-mediated signaling pathway	5	3	3	2.20E-05
			Jasmonate-mediated signaling pathway	10	9	4	6.47E-03
			WRKY family transcription factor	7	12	5	2.04E-04
			Plant-pathogen interaction	12	18	9	1.95E-03
			Cell death	4	8	3	7.90E-03
			Sugar transport	7	8	6	2.30E-04
Turquoise-Blue (448)	↓ 24 hpi	↓ 2 wpi	ABA-responsive	3	3	3	2.61E-04
			Cell wall organization and biogenesis	6	5	5	1.05E-03
			Cell growth and death	27	36	25	9.91E-12
			Regulation of cell cycle	18	22	17	2.85E-11
			Chromatin assembly	7	13	6	2.30E-04
			Cytoskeleton organization and biogenesis	4	4	4	1.60E-03
			Microtubule-driven movement	13	12	9	2.79E-05
			Myosin-driven movement	3	3	3	1.21E-03
			Beta-1,3 glucan catabolism	8	11	7	1.05E-03
			AUX/IAA family transcription factor	3	4	3	3.57E-03
			HMG family transcription factor	5	4	3	1.73E-03
			C2C2-DOF family transcription factor	3	3	3	5.98E-03
			Starch and sucrose metabolism	15	15	10	4.14E-03
			Phagosome	7	8	6	5.73E-03
Yellow-Turquoise (101)	↑ 24 hpi - 2 wpi	↑ 2 wpi	Protein kinase	21	85	15	1.48E-04
			Ethylene-mediated signaling pathway	7	18	5	1.55E-03
			Cell death	3	8	3	5.41E-04
			Starch and sucrose metabolism	4	13	4	9.72E-03
			Trehalose metabolism	2	5	2	3.21E-03
			P-type ATPase phospholipid transporting	2	2	2	7.26E-04

We also observed overlap between stem and leaf co-expression modules with similar temporal expression patterns in the two organs, such as the stem “turquoise” and leaf “yellow” modules (100 genes down-regulated at 24 hpi) and the stem “brown” and leaf “turquoise” modules (188 genes presenting an overexpression at 2 wpi). Shared genes in the stem “turquoise” and leaf “yellow” modules included 11 genes belonging to five significantly enriched photosynthesis-related FCTs and 11 others to “HSP-mediated protein folding” overrepresented FCT. Photosynthesis-associated genes have been reported to be down-regulated in plants upon challenge by both virulent and avirulent pathogens (Rojas et al., 2014), as well as in leaves of Esca-affected vines, preceding and following the appearance of foliar symptoms (Letousey et al., 2010; Magnin-Robert et al., 2011). The “Xyloglucan modification” FCT was significantly overrepresented in the genes shared by the stem “brown” and leaf “turquoise” modules. This category contained nine xyloglucan endotransglycosylase/hydrolase genes, including a genomic cluster of five adjacent genes, suggesting a possible co-regulation of these genes in response to infection. The “Peroxisome organization and biogenesis” FCT was also significantly enriched, including two catalase genes, the glycolate oxidase gene *VvGOX* (*VIT_10s0003g03830*), a xanthine dehydrogenase/oxidase and two acyl-CoA oxidase genes, as well as the “Gluthatione metabolism” with four gluthatione *S*-transferase genes, suggesting that both production and scavenging of ROS was modulated in response to *N. parvum* at 2 wpi in the two organs. Gene expression trends of stems and leaves at 24 hpi and 2 wpi in the five largest module overlaps were confirmed by RT-qPCR (*R* = 0.94; *P* < 10^−8^; Figure S5).

### Co-expression module topology and promoter motif analysis of the five largest module overlaps between local and distal responses

We analyzed the topology of the two co-expression gene networks to identify the transcription factor (TF) families that are potentially involved in the regulation of the responses to *N. parvum* in the two organs. Because intramodular hubs potentially include major transcriptional regulators of the co-expressed gene modules they belong to Ma et al. (2013), we focused on TFs that are highly connected. The two weighted networks were converted into two unweighted networks preserving all connections with a topological overlap mapping metric (TOM) > 0.1 (Data S4). Among the highly-connected genes (top 5%), 34 and 32 TF-encoding genes were found in the stem and leaf networks, respectively (Data S4). Stem and leaf hubs contained genes belonging to 10 common TF families, such as the bHLH (basic Helix-Loop-Helix), MYB (myeloblastosis), AP2/EREBP (APETALA 2/ethylene response element binding protein), WRKY, NAC [no apical meristem (NAM), ATAF1/2, cup-shaped cotyledon2 (CUC2)], and HALZ (homeobox-leucine zipper) gene families. The HALZ gene *VIT_02s0025g02590* was found among both stem “turquoise” and leaf “yellow” hubs, as well as in the corresponding module overlap, suggesting a potential regulatory role of this gene in the down-regulation of the other 99 genes in both tissues at 24 hpi. Genes encoding members of 12 other TF families were found among the stem hubs, including B3, bZIP (basic leucine zipper), GATA, and C3H zinc fingers. The TCP (TEOSINTE BRANCHED 1, CYCLOIDEA, and PCF1), HSF (heat stress factor), LIM, and bromodomain TF family genes were found among leaf hubs. Interestingly, the hubs of the leaf “turquoise” module included *VvWRKY33* (*VIT_06s0004g07500*), which was previously shown to be induced in grape leaves by both downy mildew and dehydration stress (Merz et al., 2015; Hopper et al., 2016), as well as in grape flowers by *Botrytis cinerea* (Haile et al., 2017).

We also explored the possibility that co-expressed genes in both organs, either modulated at a similar or a different time point, are regulated by common transcriptional regulators. Using the MEME motif discovery analysis (Bailey et al., 2009), we first identified recurrent DNA motifs in the promoter regions (up to 1 and 5 kb upstream of the coding sequence) of the genes belonging to the each of the five largest module overlaps. Shared motifs were then compared to known plant TF-binding motifs from the PlantTFDB v4.0 database (Jin et al., 2017; Data S5). TF-binding motif families shared by all the genes of each module overlap are provided in Data S5 Table D. Common TF-binding motifs to all genes belonging to each module overlap were found among both 1 and 5 kb upstream regions except for the genes belonging to the overlap between the stem “turquoise” and leaf “blue” modules that did not present any common annotated motif in their 1 kb upstream region. Among these common motifs, we found motifs corresponding to several TF families, such as AP2, ERF, MYB, NAC, GRAS, and zinc fingers. These results suggest that genes co-expressed, synchronously or asynchronously, in both organs might be co-regulated by common transcriptional regulators.

## Discussion

### *N. parvum* infection triggers major transcriptomic reprogramming in both stems and leaves in an asynchronous way

In this study, we show that grapevine stems and leaves undergo major transcriptional reprogramming in response to *N. parvum* infection (Figure 3B). Mapping on the combined reference transcriptome confirmed that *N. parvum* was present and transcriptionally active in stem samples, indicating that the local response at 24 hpi is the result of a direct interaction between the pathogen and the grapevine stem cells and tissues. Not surprisingly, the negligible number of reads assigned to the fungal transcriptome confirmed that *N. parvum* does not colonize leaves. Therefore, the delayed responses observed in leaves are not due to direct interaction between the pathogen and the leaf cells and tissues. We can hypothesize that leaves “perceive” infection as a result of the damage caused to the stem, potentially as reduced water conductivity due to necrosis. Alternatively, leaf responses could be: (i) activated by the perception of plant endogenous molecules produced by or in response to fungal activity, (ii) caused by phytotoxic metabolites secreted by the pathogen at the point of infection and translocated to the leaves, and/or (iii) induced as part of SAR.

Leaf responses may be due to the production and/or accumulation of molecules resulting from tissue or cellular damage, named Damage-Associated Molecular Patterns (DAMPs), and their relocation. Plants can detect the presence of pathogens through the perception of endogenous molecules, such as pectin derived oligosaccharides (PDOs) released from the plant cell wall, polypeptides/peptides produced from larger precursor proteins, extracellular ATP, and High Mobility Group Box 1-related proteins (Choi and Klessig, 2016). PDOs are oligomers of α-1,4-linked galacturonosyl residues released from plant cell walls upon partial degradation of homogalacturonan backbone by either microbial polygalacturonases during infection (Cervone et al., 1989; Cantu et al., 2008) or endogenous polygalacturonases induced by mechanical damage (Orozco-Cardenas and Ryan, 1999). We have previously reported the up-regulation of two *N. parvum* pectate lyase-encoding genes at 24 hpi in woody stems (Massonnet et al., 2016). Pectate lyase activity may contribute to the accumulation of PDOs at the point of infection (An et al., 2005). In *Arabidopsis*, a member of the Wall-Associated Kinase (WAK) family has been identified as receptor of PDOs (Brutus et al., 2010). This perception triggers several defense responses, such as ROS accumulation through the activation of the NADPH oxidase AtRbohD, nitric oxide production, callose deposition, and MAPK-mediated activation of defense gene expression (Ferrari et al., 2013). In this study, the up-regulation of seven genes encoding WAKs at 2 wpi may suggest that leaves are perceiving PDOs released in the stem. The systemic spread of fungal phytotoxins may also contribute to the activation of leaf responses, as shown in soybean affected by “sudden death syndrome” (Navi and Yang, 2008) and as proposed by Mugnai et al. (1999) in grapevines with Esca. Transcriptome dynamics during infection suggested that *N. parvum* activates co-expressed clusters of genes involved in secondary metabolism at 2 wpi (Massonnet et al., 2016). The identification of the compound(s) synthesized and secreted by *N. parvum* at 2 wpi *in planta*, as well as the breakdown products of the plant and fungal cell walls, will help shed light on the role of toxins and DAMPs in the expression of disease symptoms in distal parts of the plant.

Leaf responses could also be due in part to SAR. In plants, distal tissues can activate defenses to a broad-spectrum of pathogens in response to local infection (Conrath, 2006). This phenomenon depends on an effective long-distance communication between plant organs, relying on the generation and transport of signals (Shah and Zeier, 2013; Gao et al., 2015). These signals lead to the production of antimicrobial compounds in distal tissues that protect the rest of the plant from secondary infections (Durrant and Dong, 2004). In *Arabidopsis*, the onset of SAR is dependent on the transcription cofactor NPR1 (Nonexpressor of PR genes) and its associated TFs, such as TGAs (Fu and Dong, 2013). In our study, *VvNPR1.1* (*VIT_11s0016g01990*; Le Henanff et al., 2011) was up-regulated at 24 hpi in stems and at both 24 hpi and 2 wpi in leaves, suggesting that the distal response could be associated with activation of SAR, which started at 24 hpi and was fully established by 2 wpi. Further experiments are necessary to establish if broad-spectrum defenses are activated in distal tissues in response to *N. parvum* infection. In addition, the up-regulation at 2 wpi of the two key TF-encoding genes previously characterized to be involved in biotic stresses in grape leaves *VvWRKY2* (*VIT_01s0011g00220*; Mzid et al., 2007) and *VvNAC1* (*VIT_08s0007g07670*; Le Henanff et al., 2013) suggests that the distal response to *N. parvum* infection consists of a combination of multiple stress responses.

### The temporal shift of the response was characterized by numerous co-expressed genes involved in several plant defense-related mechanisms

Co-expression network analysis showed that both local and distal responses involved the co-expression of common sets of genes differentially regulated at 24 hpi in stems and 2 wpi in leaves. These common, but asynchronous, transcriptomic rearrangements affected genes involved in both signal perception and transduction, as well as several downstream biological processes. The asynchronous co-induction of the alternative NAD(P)H dehydrogenase gene *VvaND1* and the alternative oxidase (AOX) gene *VvAOX23* in both tissues at 24 hpi in stems and at 2 wpi in leaves suggests that the two organs undergo oxidative stress at different time points after infection (Vanlerberghe, 2013). Alternative NAD(P)H dehydrogenases and AOXs are part of a non-phosphorylating respiratory pathway, the role of which is to prevent over-reduction of the mitochondrial respiratory chain and to help balance cellular redox levels in response to cellular stress (Van Aken et al., 2009). Several studies have shown that mitochondrial dysfunctions, often associated with oxidative stress, result in the induction of AOX at the transcript and protein level, thus making AOX a general marker of mitochondrial dysfunction and/or cellular oxidative stress (Vanlerberghe, 2013). At the cellular level, oxidative stress can cause damage to several biomolecules, such as lipids, proteins and DNA. These reactions can alter intrinsic membrane properties like fluidity, ion transport, loss of enzyme activity, protein cross-linking, inhibition of protein synthesis, DNA damage, and ultimately lead to cell death (Khan et al., 2017). In wood, oxidative stress might promote lignin polymerization in the apoplast, potentially as a defense mechanism against pathogen colonization (Vanholme et al., 2010). In leaves, processes associated with oxidative stress may contribute to leaf scorching and senescence (Sedigheh et al., 2011), symptoms we found in leaves of IW plants.

In addition, the significant overrepresentation of the FCTs “Regulation of the cell cycle” and “Microtubule organization and biogenesis” among the stem “blue”—leaf “turquoise” module overlap, corresponding to genes presenting a down-regulation at 24 hpi in stems and 2 wpi in leaves (stem “turquoise”—leaf “blue” module overlap), suggests an inhibition of cell proliferation in both organs in response to *N. parvum* colonization, while the enriched FCT “Cell wall organization and biogenesis” including the two α-expansin genes *VvEXPA6* (*VIT_06s0004g04860*) and *VvEXPA18* (*VIT_17s0053g00990*) indicate that also cell expansion is affected in diseased vines (Rose et al., 1997; Cosgrove, 2000). Both biotic and abiotic stresses are known to negatively affect plant growth through inhibition of the cell-cycle machinery (De Veylder et al., 2007). In addition, treatment with pathogen-derived molecules have been showed to trigger the down-regulation of some cell cycle-related genes (Suzuki et al., 2006; Kawaguchi et al., 2012). An extensive alteration of cell cycle in the infected vines is also supported by the significant enrichment in the “Cell death” FCT among the stem “blue”—leaf “turquoise” module overlap, which included two genes encoding proteins containing a membrane-attack complex/perforin (MACPF) domain (*VIT_01s0011g05950*; *VIT_05s0062g00790*). MACPF domains were reported to be negative regulators of the cell death programs and defense responses in *Arabidopsis* (Morita-Yamamuro et al., 2005; Noutoshi et al., 2006).

### Local-specific response during *N. parvum* infection

Differential gene expression analysis identified a greater number of DEGs in the stems compared to the leaves, suggesting a more intense response at the transcriptomic level at the point of infection than in distal tissue. This phenomenon has been also observed in other plant biotic interactions (Babst et al., 2009; Zhang et al., 2012; Ling et al., 2015). In our study the more intense transcriptional reprogramming in stem is likely due to the direct and continuous interaction between the stem and the pathogen during the experiment time course. The colonization of the woody tissue by *N. parvum* and consequent canker development is due to extensive decomposition of the plant cell walls, damage to the vascular tissue, and necrosis of the living cells in the organ (Rolshausen et al., 2008; Galarneau et al., 2016). Oxidative burst and release of ROS in the tissues attacked by the pathogen (Camejo et al., 2016) may explain the overexpression of more numerous genes involved in ROS scavenging in the stems compared to the leaves. This strong oxidative stress in the woody stem may be associated with the induction of the citric acid cycle at 24 hpi, suggested by the significant up-regulation of genes encoding proteins of the cycle (Mailloux et al., 2007; Avin-Wittenberg et al., 2012). Plant respiration is known to be stimulated during biotic stress (Bolton, 2009), as well as the up-regulation of citric acid cycle-associated genes (Less et al., 2011). The induction of these genes affects downstream defense responses, such as the generation of ROS and the activation of PR genes (Rojas et al., 2014).

Lignin and stilbene biosynthesis were among the pathways exclusively activated in the stem. Increased lignification of the tissue during the interaction may be interpreted as an attempt of the plant to reinforce and/or repair the plant cell walls affected by *N. parvum* colonization, thus limiting further spread of the pathogen (Smith et al., 2007; Miedes et al., 2014). Stilbene biosynthesis is also a widely deployed inducible defense mechanism in plants (Chong et al., 2009). Stilbenes act as antimicrobial compound and at the same time participate to scavenging the ROS that accumulate during the interaction with the pathogen (Teguo et al., 1998; Morales et al., 2000; Jeandet et al., 2002). In grapevine, the induction of STS genes and the accumulation of stilbene compounds have been reported in many studies focused on both biotic and abiotic stresses (Bézier et al., 2002; Wang et al., 2010; Malacarne et al., 2011; Vannozzi et al., 2012; Amrine K. C. H. et al., 2015). Stilbene accumulation was also found in grapevine trunks with Botryosphaeria dieback symptoms. The absence of an induction of stilbene biosynthesis in leaves during *N. parvum* infection confirmed previous observations in the same cultivar we used in this study, “Cabernet Sauvignon” (Bellée et al., 2016). The up-regulation of STS genes in leaves of “Merlot” and “Ugni-blanc” indicates that grapevine responses to *N. parvum* may be different in the different genetic backgrounds.

## Conclusions

This work begins to highlight the underlying complexity of the systemic responses to a trunk pathogen in grapevines. The identified responses in stems and leaves provide a first glimpse of the functions activated in the two organs and their temporal regulation. We cannot rule out that the patterns observed in this study are specific to “Cabernet Sauvignon.” Variability in susceptibility to trunk pathogens has been described (Bruez et al., 2013; Travadon et al., 2013; Murolo and Romanazzi, 2014). As similar studies are carried out in more genotypes, associations between level of tolerance/susceptibility to a trunk pathogen and gene expression can be determined. This information not only can help dissect the molecular bases of the interaction, but can also be incorporated in breeding programs that aim to reduce grape susceptibility to trunk pathogens. Our results suggest that leaves perceive pathogen colonization of the stem and activate responses that partially overlap with those activated at the site of infection. Topology analysis of each gene co-expression modules pointed out several highly-connected genes encoding transcriptional factors that are potentially involved in the regulation of their corresponding co-expression module members. Promoter motif analysis of the co-expressed genes modulated at different time points during *N. parvum* colonization suggests that an asynchronous co-regulation might be at the origin of the temporal shift of transcriptional reprogramming between the two organs. Understanding the signals that are responsible for the communication between different grapevine organs during infection will help shed light into the systemic signaling mechanisms in woody plants. The identification of molecular patterns that accumulate in leaves specifically in presence of trunk infection will enable early detection of trunk diseases and the timely removal of infected parts.

## Author contributions

KB and DC conceived the study. EG, DL, and KB carried out the plant experiment. RF performed the RNA extraction and RNAseq libraries. QS carried out the microscopy. CW performed the metabolite profiling assays and EG analyzed the generated results. MM carried out the computational analysis. SM and MM conducted the RT-qPCR experiments. MM and DC wrote the manuscript. All authors read and approved the final manuscript.

### Conflict of interest statement

The authors declare that the research was conducted in the absence of any commercial or financial relationships that could be construed as a potential conflict of interest.
