# Supplementary material for: Neofusicoccum parvum Colonization of the Grapevine Woody Stem Triggers Asynchronous Host Responses at the Site of Infection and in the Leaves
Source: Front Plant Sci. 2017 Jun 28;8:1117. doi: 10.3389/fpls.2017.01117 (PMC5487829; doi:10.3389/fpls.2017.01117)
Supplement: Supplementary file 10 [file Image1.PDF]

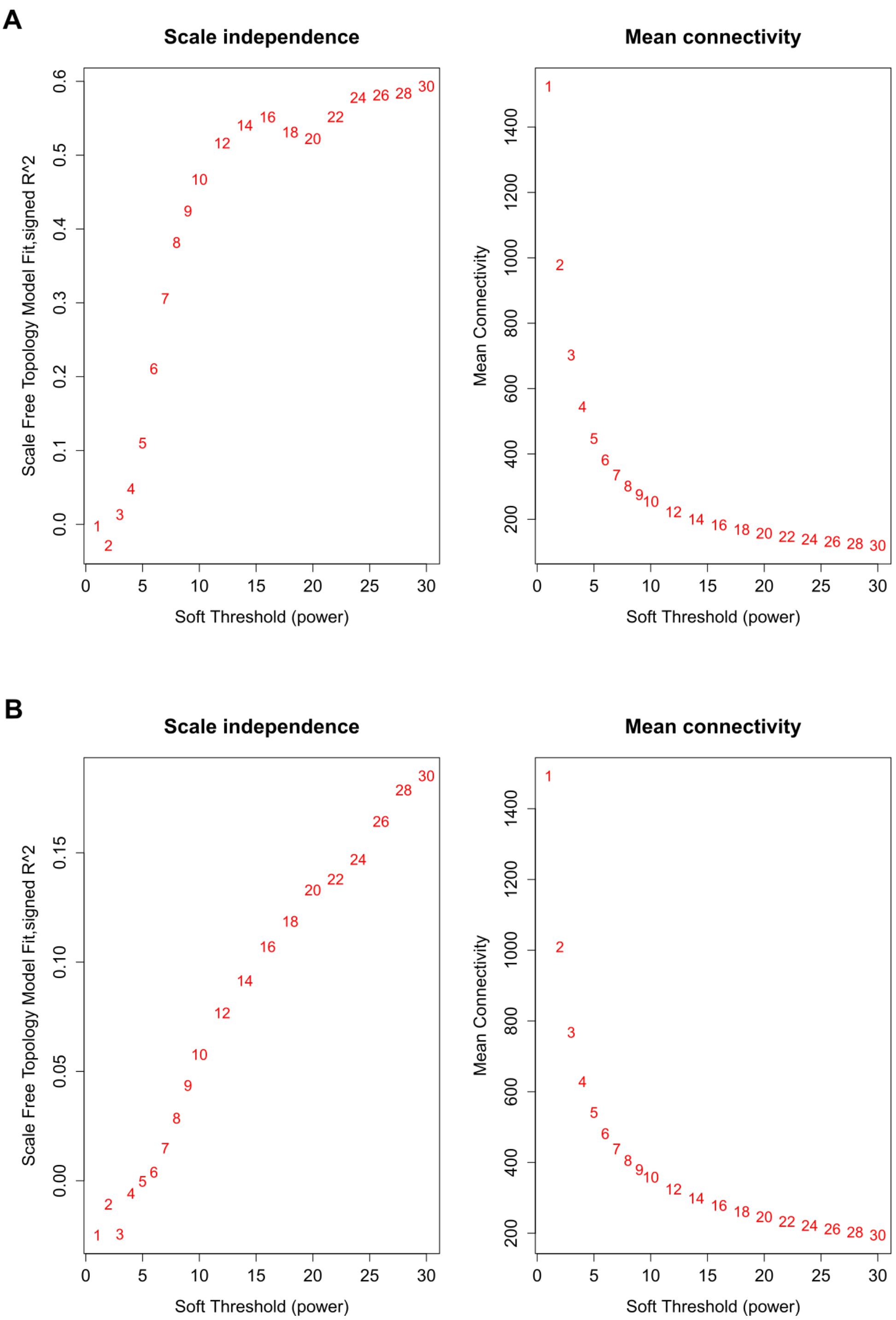

Figure S1: Analysis of network topology for various soft-thresholding powers among stem (**A**) and leaf (**B**) samples. The left panel shows the scale-free fit index (y-axis) as a function of the soft-thresholding power (x-axis). The right panel displays the mean connectivity (degree, y-axis) as a function of the soft-thresholding power (x-axis). This analysis was processed on the log_2-FC values (IW/NINW) of the 2,978 DEGs shared by both local and distal responses at each time point.
