# Supplementary material for: Neofusicoccum parvum Colonization of the Grapevine Woody Stem Triggers Asynchronous Host Responses at the Site of Infection and in the Leaves
Source: Front Plant Sci. 2017 Jun 28;8:1117. doi: 10.3389/fpls.2017.01117 (PMC5487829; doi:10.3389/fpls.2017.01117)
Supplement: Supplementary file 11 [file Image2.PDF]

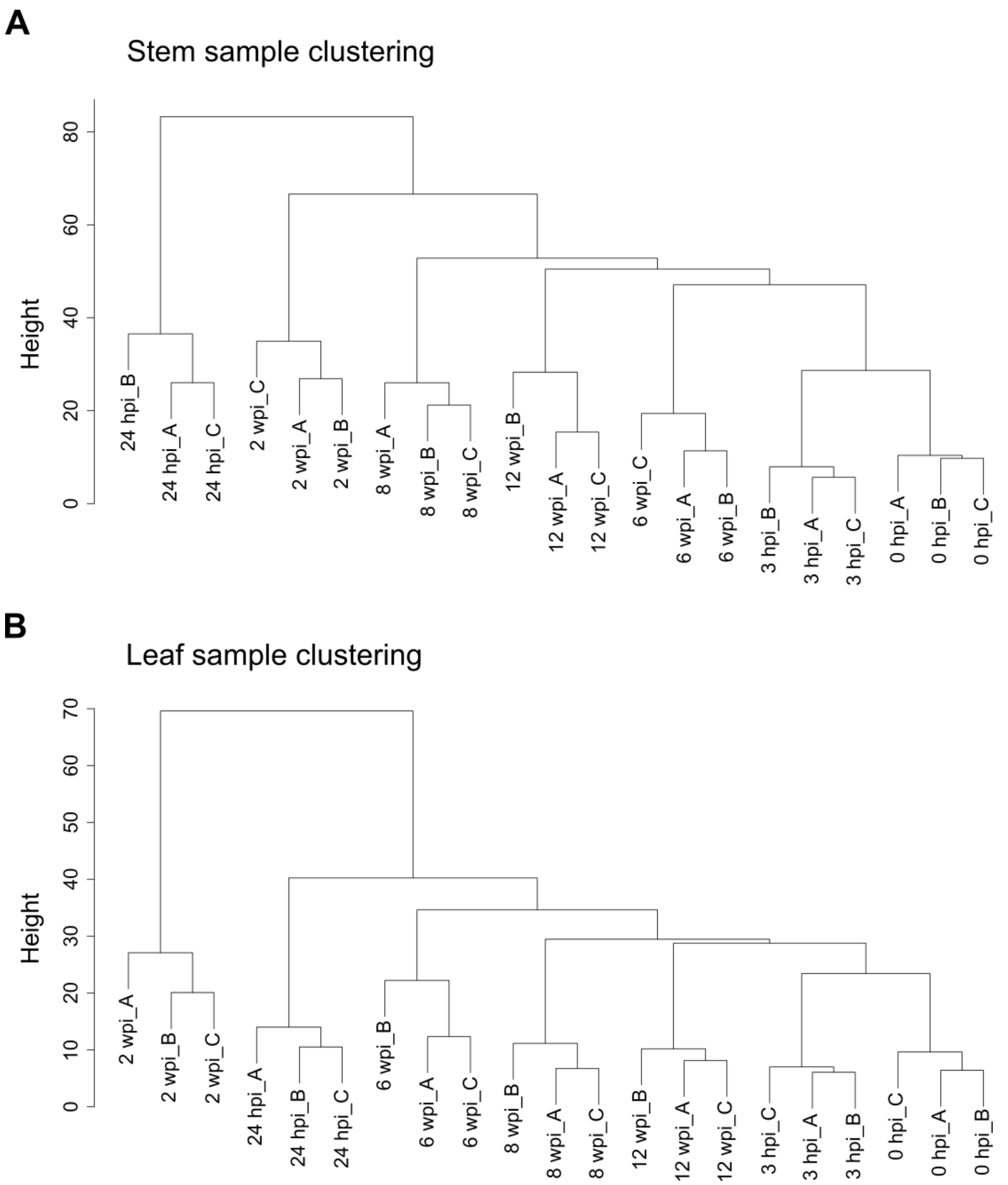

Figure S2: Clustering dendrograms of stem (**A**) and leaf (**B**) samples based on their Euclidean distance. This analysis was processed on the log_2-FC values (IW/NINW) of the 2,978 DEGs shared by both local and distal responses at each time point.
