# Supplementary material for: Neofusicoccum parvum Colonization of the Grapevine Woody Stem Triggers Asynchronous Host Responses at the Site of Infection and in the Leaves
Source: Front Plant Sci. 2017 Jun 28;8:1117. doi: 10.3389/fpls.2017.01117 (PMC5487829; doi:10.3389/fpls.2017.01117)
Supplement: Supplementary file 12 [file Image3.PDF]

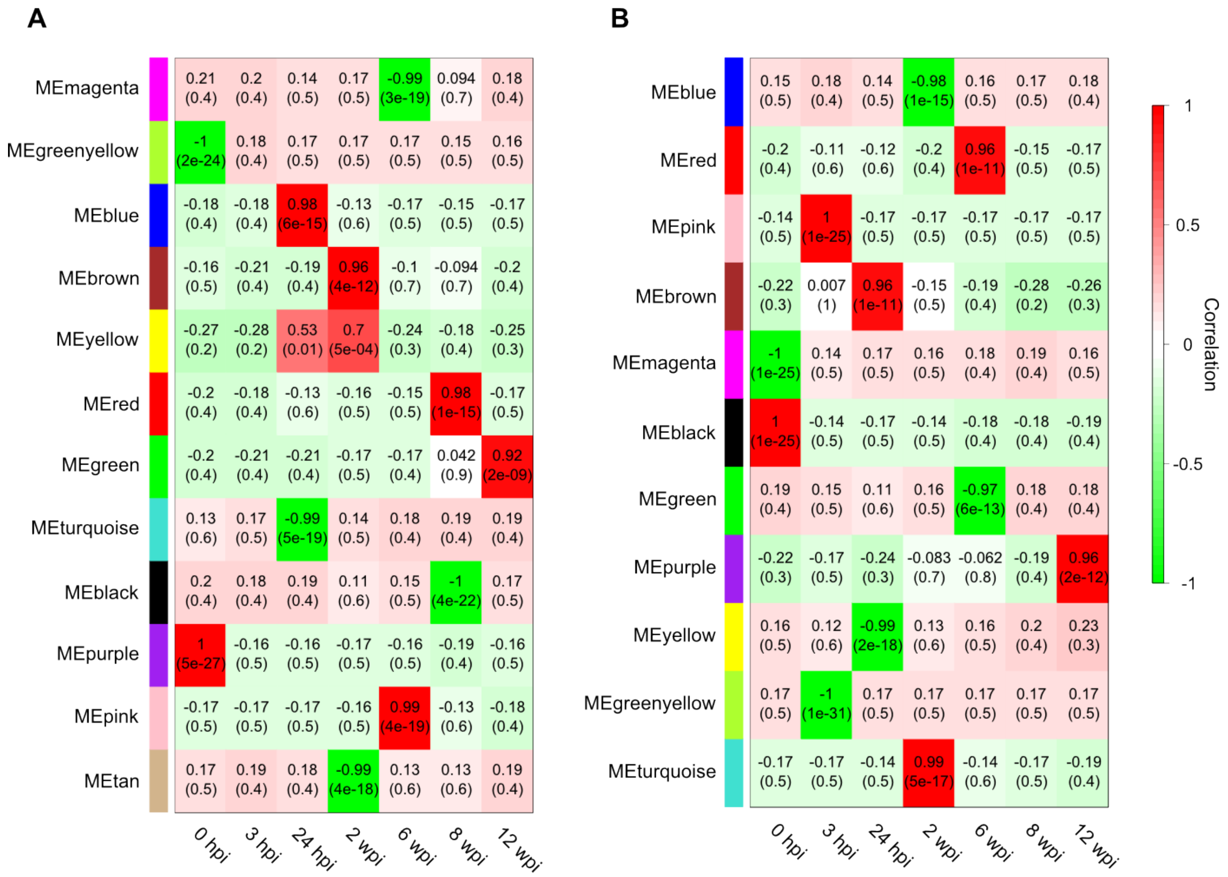

Figure S3: Module-trait (time point) associations in stem (**A**) and leaf (**B**) networks. Each row corresponds to a module eigengene and each column to a time point. Each cell contains the corresponding correlation and *P*-value. The table is color-coded by correlation according to the color legend.
