# Supplementary material for: Neofusicoccum parvum Colonization of the Grapevine Woody Stem Triggers Asynchronous Host Responses at the Site of Infection and in the Leaves
Source: Front Plant Sci. 2017 Jun 28;8:1117. doi: 10.3389/fpls.2017.01117 (PMC5487829; doi:10.3389/fpls.2017.01117)
Supplement: Supplementary file 13 [file Image4.PDF]

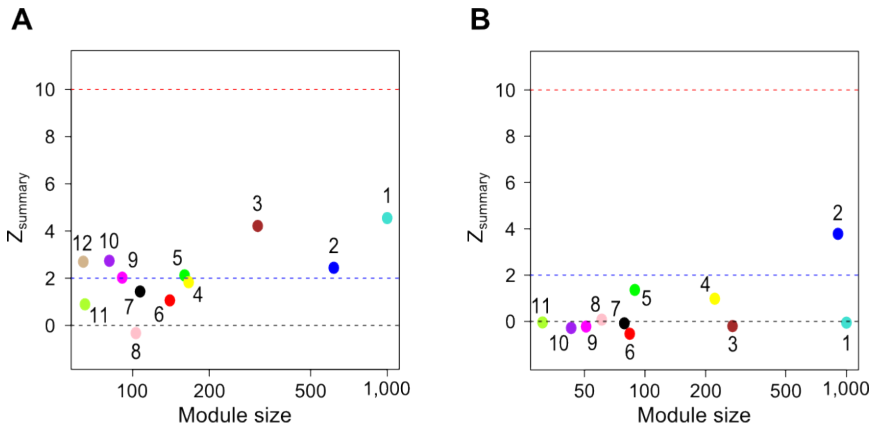

Figure S4: Composite preservation statistics of stem modules in leaf samples (**A**) and vice versa (**B**). The overall significance of the preservation statistics was assessed using $Z_{summary}$ that combines multiple preservation statistics into a single overall measure of preservation (Langfelder *et al.*, 2011). According Langfelder *et al.* (2011), if $Z_{summary} > 10$ there is strong evidence that the module is preserved; if $2 < Z_{summary} < 10$ there is weak to moderate evidence of preservation; if $Z_{summary} < 2$, there is no evidence that the module is preserved. (**A**) The summary statistic $Z_{summary}$ (y-axis) of stem modules as a function of the module size. Each point represents a module, labelled by color and a secondary numeric label (1=turquoise, 2=blue, 3=brown, 4=yellow, 5=green, 6=red, 7=black, 8=pink, 9=magenta, 10=purple, 11=greenyellow, 12=tan). (**B**) The summary statistic of leaf modules as a function of the module size. Each point represents a module, labelled by color and a secondary numeric label (1=turquoise, 2=blue, 3=brown, 4=yellow, 5=green, 6=red, 7=black, 8=pink, 9=magenta, 10=purple, 11=greenyellow). The dashed blue and red lines indicate the thresholds Z=2 and Z=10, respectively.
