# Supplementary material for: Neofusicoccum parvum Colonization of the Grapevine Woody Stem Triggers Asynchronous Host Responses at the Site of Infection and in the Leaves
Source: Front Plant Sci. 2017 Jun 28;8:1117. doi: 10.3389/fpls.2017.01117 (PMC5487829; doi:10.3389/fpls.2017.01117)
Supplement: Supplementary file 14 [file Image5.PDF]

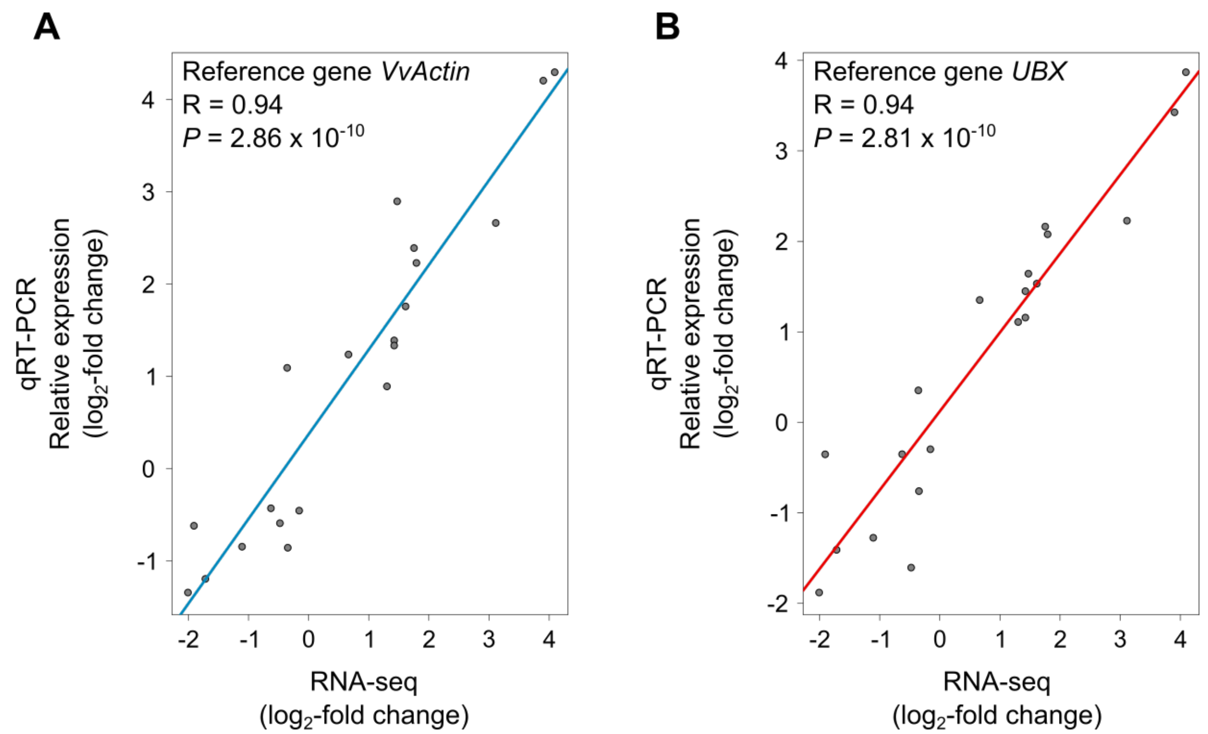

Figure S5: Scatterplots showing the correlation between the fold changes (log_2) in expression obtained by RNAseq and the fold changes (log_2) measured by qRT-PCR. Relative expression was calculated using *VvActin* (**A**) and a UBX-domain-containing protein gene (*UBX*) (**B**) as reference genes. Linear trends, correlation coefficient factors (R) and *P*-values are provided.
