# Supplementary material for: Neofusicoccum parvum Colonization of the Grapevine Woody Stem Triggers Asynchronous Host Responses at the Site of Infection and in the Leaves
Source: Front Plant Sci. 2017 Jun 28;8:1117. doi: 10.3389/fpls.2017.01117 (PMC5487829; doi:10.3389/fpls.2017.01117)
Supplement: Supplementary file 15 [file Image6.PDF]

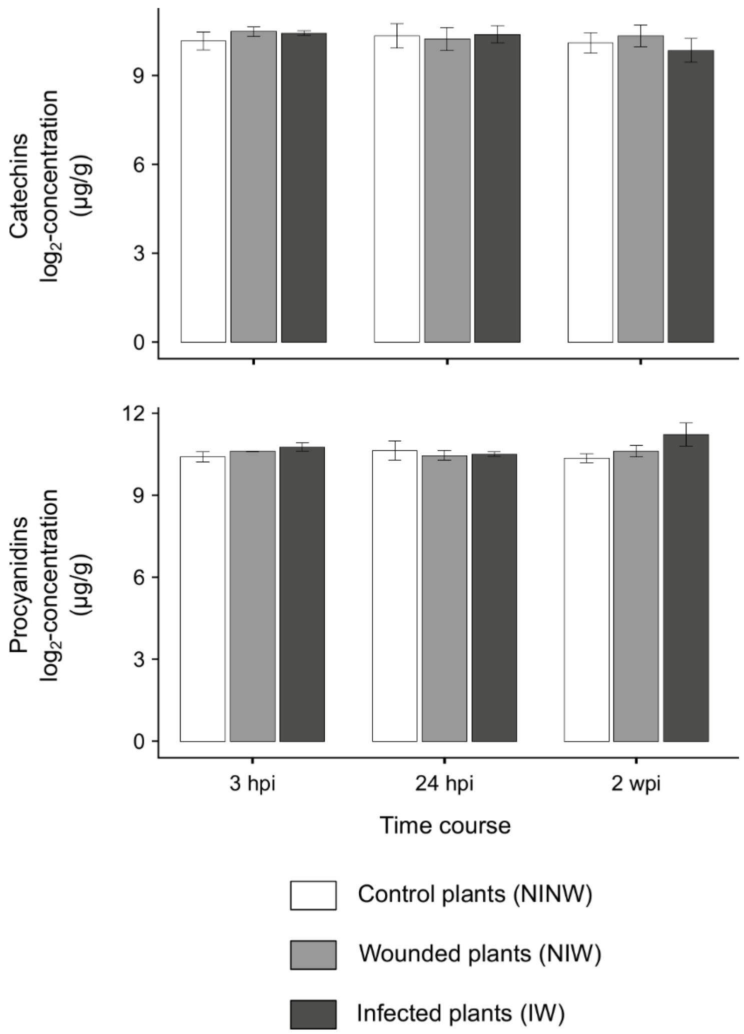

Figure S6: Catechins and procyanidins compounds concentration profiling in woody stems. Catechins and procyanidins concentrations in stems were determined at three time points post-inoculation by HPLC assays. Data are expressed as mean ± standard deviation. ANOVA with Tukey's post hoc test was used to compare the $\log_2$-transformed concentrations between the three treatment conditions (IW, NIW, NINW) at each time point. Any comparison was considered statistically significant (adjusted $P < 0.05$).
