# Supplementary material for: Neofusicoccum parvum Colonization of the Grapevine Woody Stem Triggers Asynchronous Host Responses at the Site of Infection and in the Leaves
Source: Front Plant Sci. 2017 Jun 28;8:1117. doi: 10.3389/fpls.2017.01117 (PMC5487829; doi:10.3389/fpls.2017.01117)
Supplement: Supplementary file 17 [file Image8.PDF]

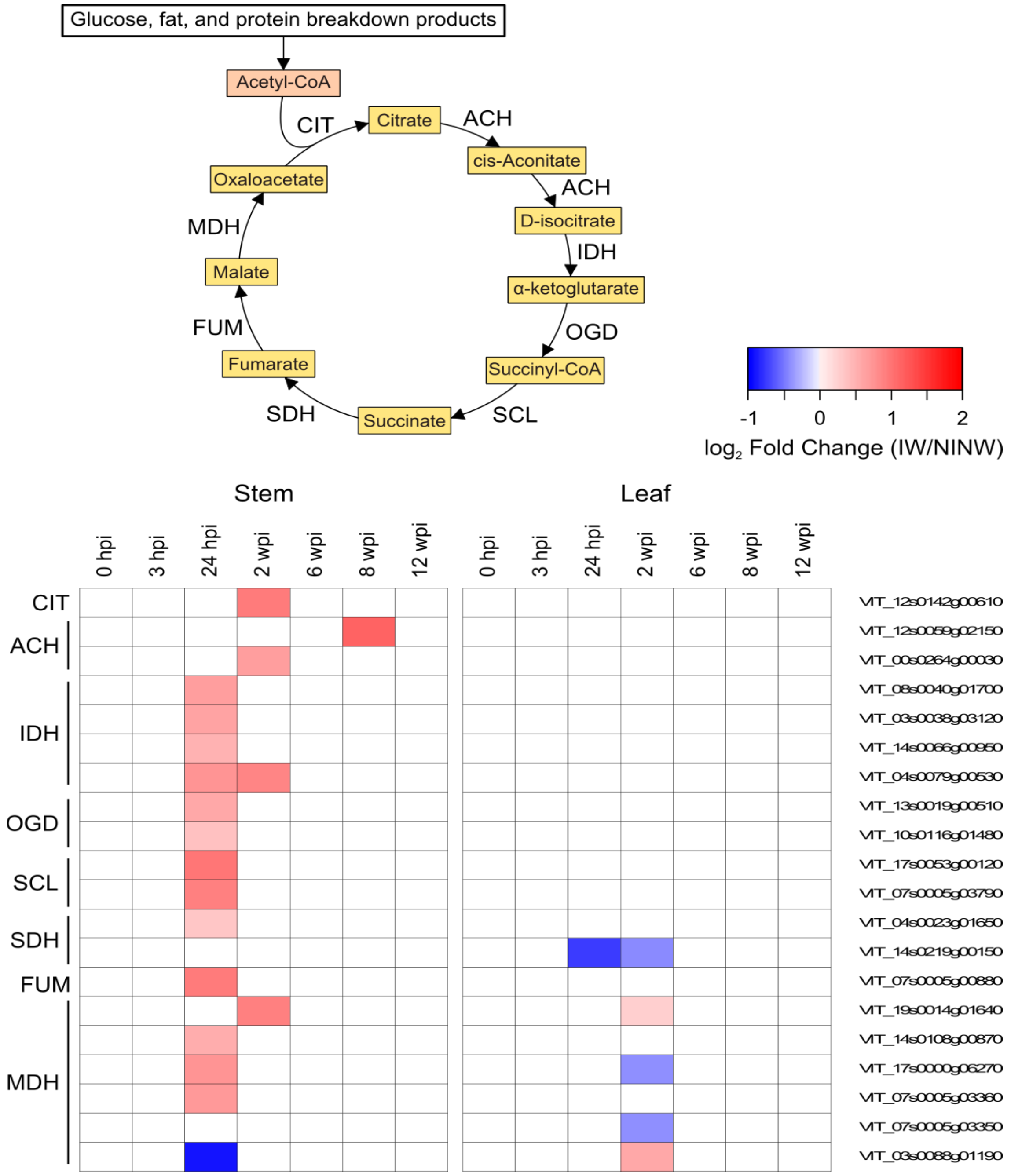

Figure S8: Heat map of the citric acid cycle-associated genes which expression was modulated during *N. parvum* infection. CIT, Citrate synthase; ACH, Aconitate hydratase; IDH, Isocitrate dehydrogenase; OGD, 2-oxoglutarate dehydrogenase; SCL, Succinyl-CoA ligase; SDH, Succinate dehydrogenase; FUM, Fumarate hydratase; MDH, Malate dehydrogenase.
