# Supplementary material for: Neofusicoccum parvum Colonization of the Grapevine Woody Stem Triggers Asynchronous Host Responses at the Site of Infection and in the Leaves
Source: Front Plant Sci. 2017 Jun 28;8:1117. doi: 10.3389/fpls.2017.01117 (PMC5487829; doi:10.3389/fpls.2017.01117)
Supplement: Supplementary file 18 [file Image9.PDF]

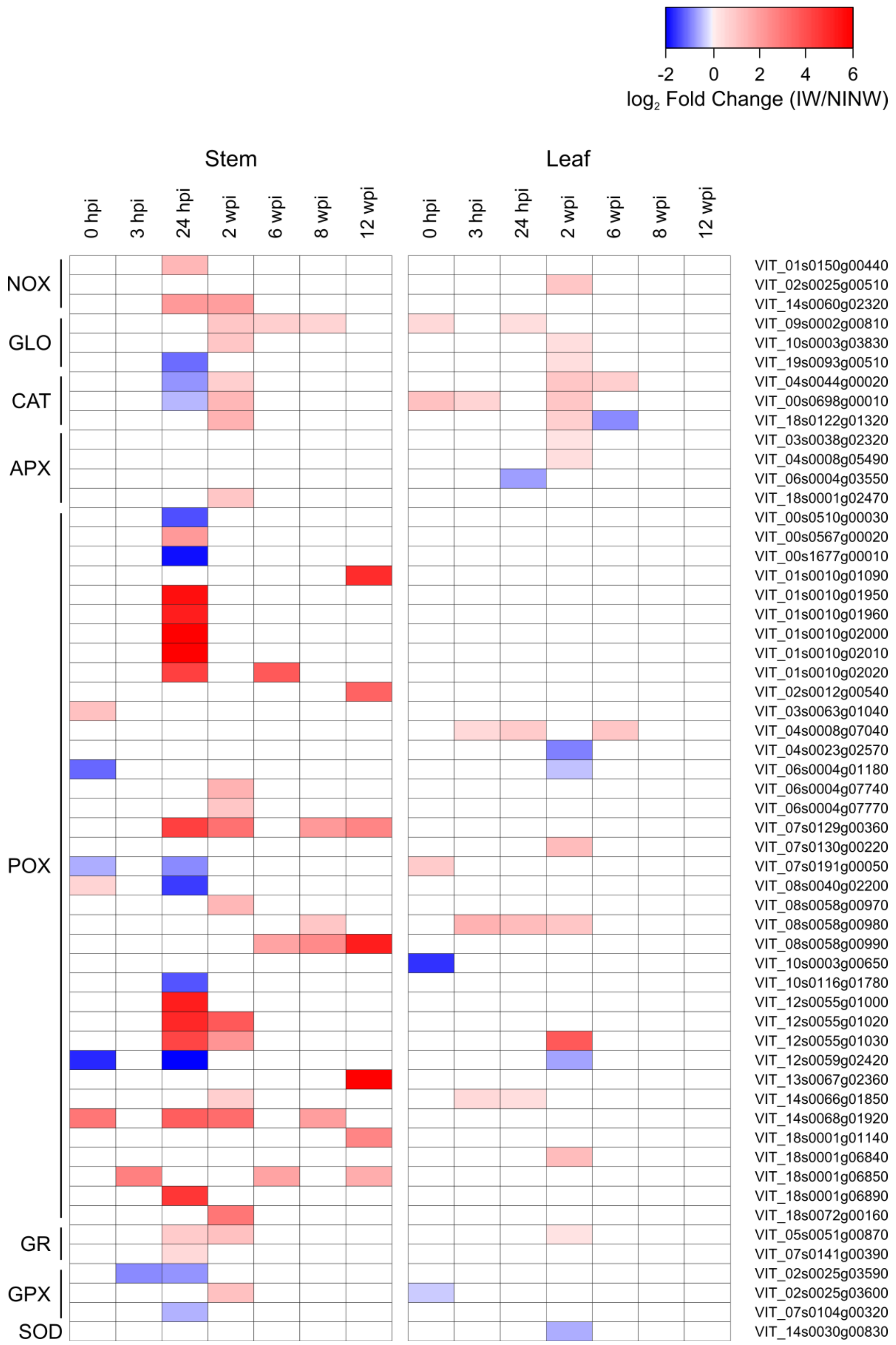

Figure S9: Heat map of ROS production- and scavenging-associated genes whose expression was modulated during *N. parvum* infection. NOX, NADPH oxidase; GLO, peroxisomal S-2-hydroxy-acid oxidase; CAT, Catalase; APX, L-ascorbate peroxidase; POX, Class III peroxidase; GR, Glutathione reductase; GPX, Glutathione peroxidase; SOD, Cu/Zn superoxide dismutase.
