# Supplementary material for: Neofusicoccum parvum Colonization of the Grapevine Woody Stem Triggers Asynchronous Host Responses at the Site of Infection and in the Leaves
Source: Front Plant Sci. 2017 Jun 28;8:1117. doi: 10.3389/fpls.2017.01117 (PMC5487829; doi:10.3389/fpls.2017.01117)
Supplement: Supplementary file 19 [file Image10.PDF]

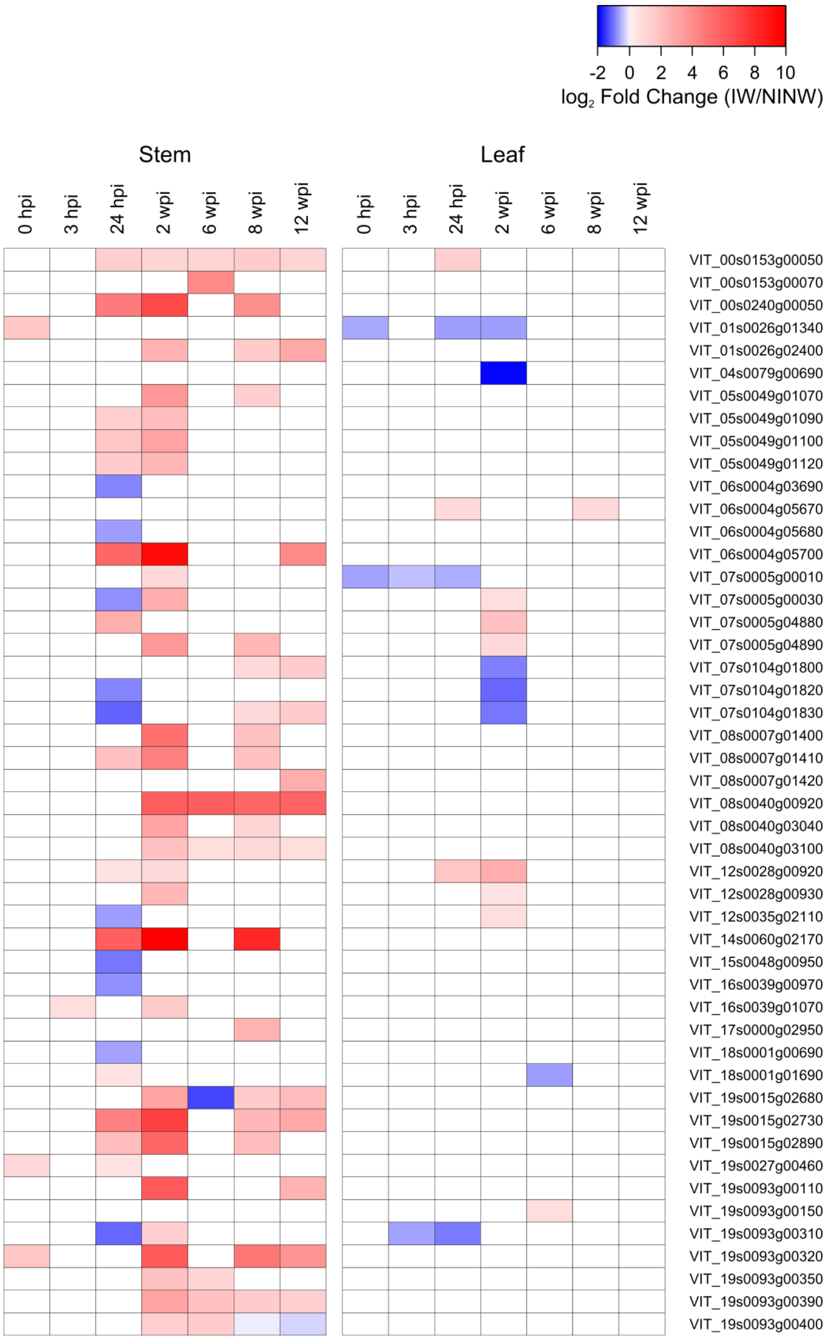

Figure S10: Heat map of the glutathione *S*-transferase (GST)-encoding genes identified as differentially expressed in response to *N. parvum* infection.
