# Supplementary material for: Neofusicoccum parvum Colonization of the Grapevine Woody Stem Triggers Asynchronous Host Responses at the Site of Infection and in the Leaves
Source: Front Plant Sci. 2017 Jun 28;8:1117. doi: 10.3389/fpls.2017.01117 (PMC5487829; doi:10.3389/fpls.2017.01117)
Supplement: Supplementary file 20 [file Image11.PDF]

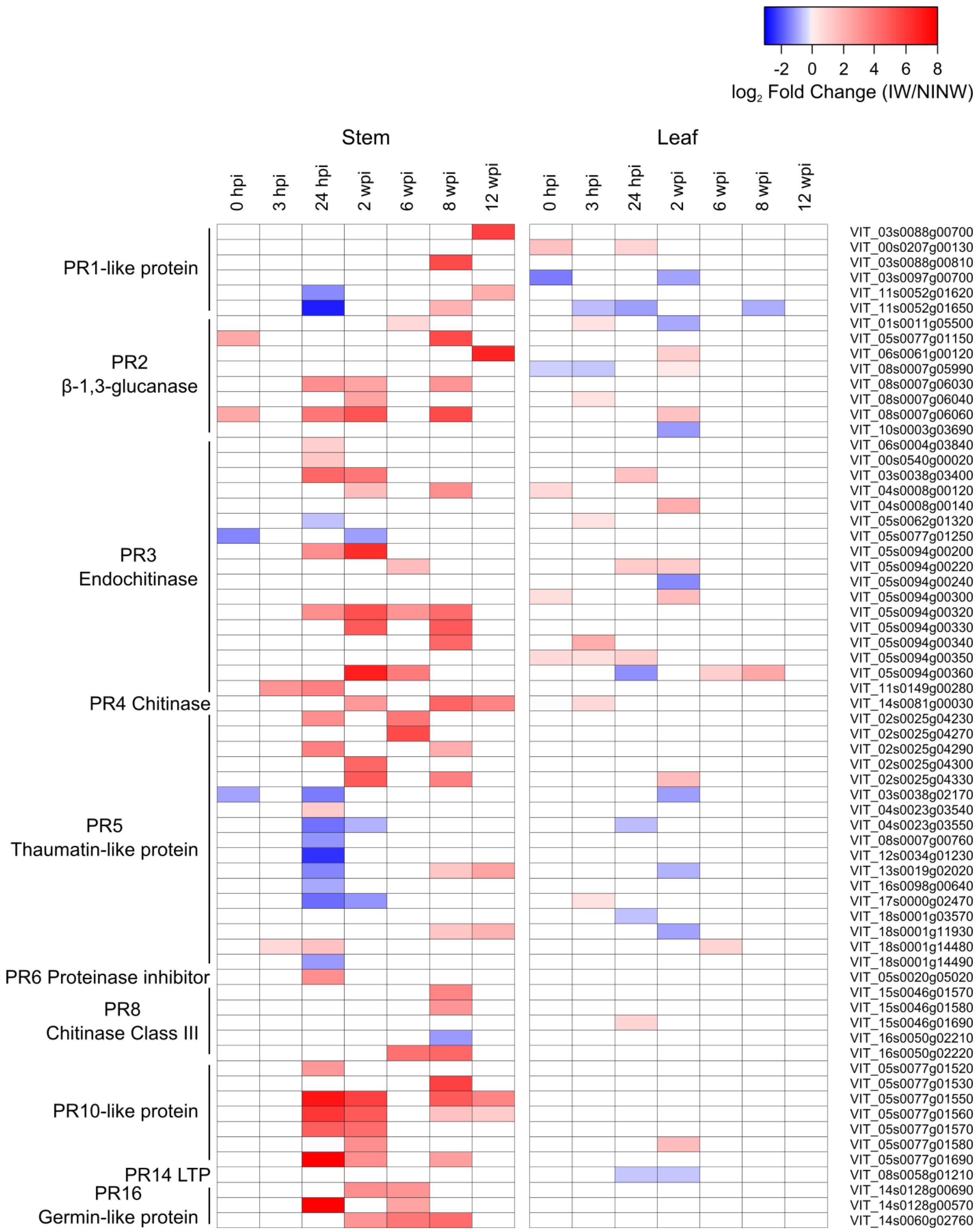

Figure S11: Heat map of the main pathogenesis-related (PR) protein-encoding genes differentially regulated in grapevine during *N. parvum* colonization. LTP, Lipid transfer protein.
