# Supplementary material for: Neofusicoccum parvum Colonization of the Grapevine Woody Stem Triggers Asynchronous Host Responses at the Site of Infection and in the Leaves
Source: Front Plant Sci. 2017 Jun 28;8:1117. doi: 10.3389/fpls.2017.01117 (PMC5487829; doi:10.3389/fpls.2017.01117)
Supplement: Supplementary file 21 [file Image12.PDF]

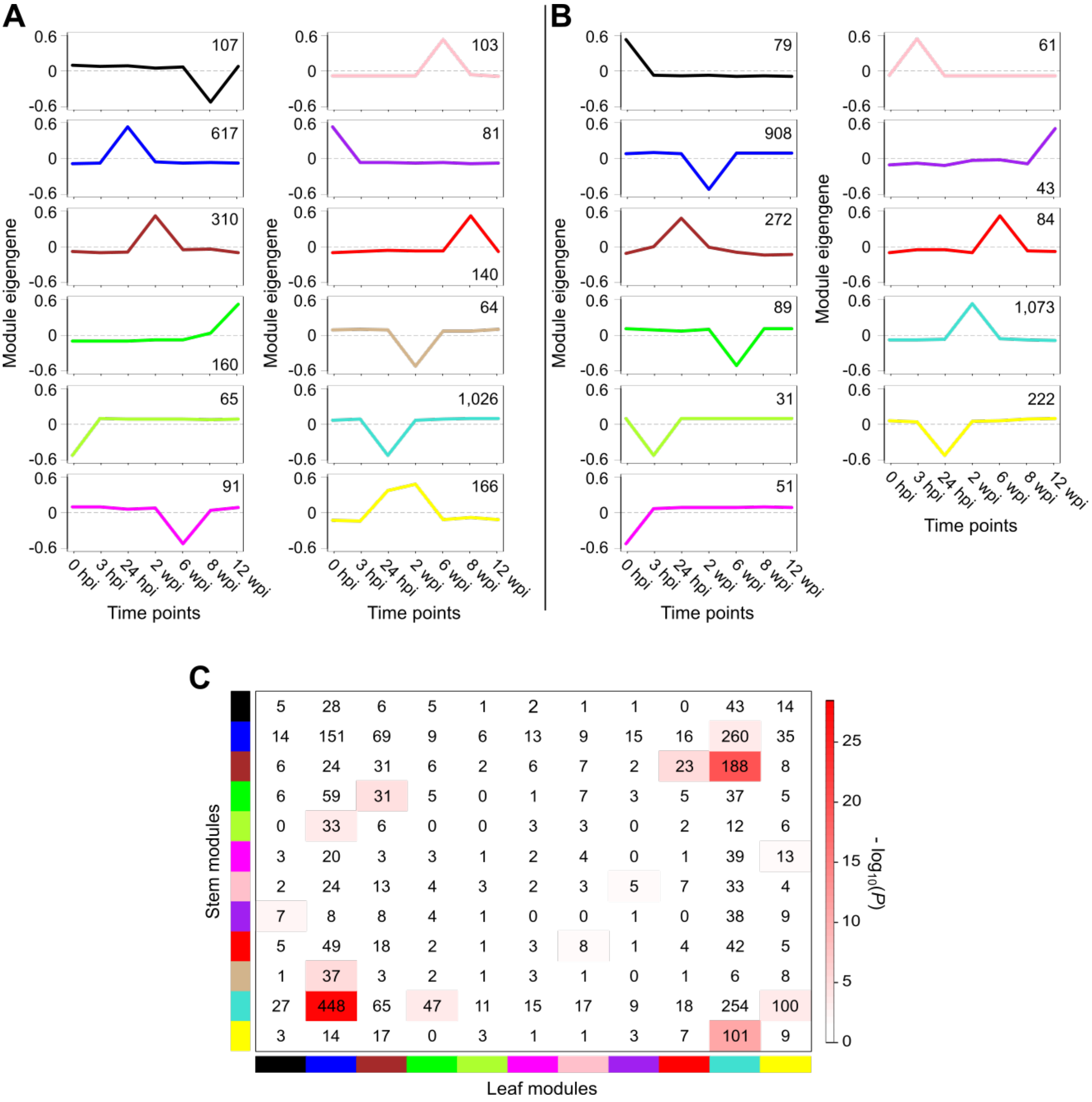

Figure S12: Comparison of stem and leaf co-expression modules. (**A**) Module eigengenes in stem network. (**B**) Module eigengenes in leaf network. The eigengene can be interpreted as a weighted average gene expression profile (Langfelder *et al*., 2011). For each module, the eigengene expression profile is color-coded as the corresponding module color and the module size is indicated. (**C**) Cross-tabulation of stem (rows) and leaf modules (columns). Each row and column is represented by the corresponding module color. In the table, numbers give counts of genes in the intersection of the corresponding row and column module. The table is color-coded by -log(*P*), the Fisher exact test *P* value, according to the color legend on the right.
